# Multivesicular Liposomes for Glucose-Responsive Insulin Delivery

**DOI:** 10.3390/pharmaceutics14010021

**Published:** 2021-12-22

**Authors:** Guangqu Liu, Suping He, Yu Ding, Cai Chen, Qingchun Cai, Wei Zhou

**Affiliations:** Department of Pharmaceutics, China Pharmaceutical University, Nanjing 211198, China; 1731010029@stu.cpu.edu.cn (G.L.); 3220010100@stu.cpu.edu.cn (S.H.); 1631010032@stu.cpu.edu.cn (Y.D.); 3320011147@stu.cpu.edu.cn (C.C.); 3319010277@stu.cpu.edu.cn (Q.C.)

**Keywords:** MVL, glucose-responsive, insulin, in situ catalysis, Alizarin red probe, molecular docking

## Abstract

An intelligent insulin delivery system is highly desirable for diabetes management. Herein, we developed a novel glucose-responsive multivesicular liposome (MVL) for self-regulated insulin delivery using the double emulsion method. Glucose-responsive MVLs could effectively regulate insulin release in response to fluctuating glucose concentrations in vitro. Notably, in situ released glucose oxidase catalyzed glucose enrichment on the MVL surface, based on the combination of (3-fluoro-4-((octyloxy)carbonyl)phenyl)boronic acid and glucose. The outer MVL membrane was destroyed when triggered by the local acidic and H_2_O_2_-enriched microenvironment induced by glucose oxidase catalysis in situ, followed by the further release of entrapped insulin. Moreover, the Alizarin red probe and molecular docking were used to clarify the glucose-responsive mechanism of MVLs. Utilizing chemically induced type 1 diabetic rats, we demonstrated that the glucose-responsive MVLs could effectively regulate blood glucose levels within a normal range. Our findings suggest that glucose-responsive MVLs with good biocompatibility may have promising applications in diabetes treatment.

## 1. Introduction

Diabetes mellitus is a chronic metabolic disease characterized by hyperglycemia, which is considered a serious threat to human health [1,2,3]. Long-standing hyperglycemia is known to cause several complications, including blindness, cerebrovascular disease, and kidney failure [4]. Insulin is the only hypoglycemic hormone in the human body and is essential for the treating patients with diabetes to reduce blood glucose levels (BGLs) [5]. However, inadequate or excessive insulin injection can result in poor glycemic control [6]. Compared with traditional insulin delivery systems, closed-loop insulin delivery systems can effectively regulate BGLs within the normal range. This type of system can discharge sufficient insulin during hyperglycemia and self-adjust to release a smaller insulin dose during normoglycemia; this is desirable for improving the quality of life of patients with diabetes [7].

Notably, to construct a closed-loop insulin delivery system, glucose-sensitive elements need to be employed; these mainly include phenylboronic acid (PBA)-containing polymers, glucose oxidase (GOx), concanavalin A, and modified insulins [4]. PBA can reversibly form boronic acid–diol complexes with 1,2- or 1,3-diols via covalent bonds [8,9]. Accordingly, PBA-containing polymers were designed and synthesized by chemical reactions, which were further used to construct glucose-responsive systems, including microcapsules [10], microspheres [11], gels [12], and micelles [13]. In these systems, the structures were destroyed when PBA combined with glucose under high glucose conditions, and the entrapped drugs were further released. However, it is difficult to synthesize PBA-containing polymers with high molecular weights, and their quality cannot be controlled. Conversely, PBA derivatives with low molecular weights are easily obtained and stable in quality, which could be employed to construct a new glucose-responsive system. GOx catalyzes the rapid and efficient conversion of glucose into gluconic acid and hydrogen peroxide (H_2_O_2_), accompanied by oxygen consumption. Thus, an environment composed of low pH, H_2_O_2_, or hypoxia offers strategies for constructing a glucose-responsive system [14,15,16,17]. Concanavalin A, known to possess high specificity and affinity for glucose, can form a three-dimensional cross-linked network structure with a glucose-containing polymer. The network structure of the insulin delivery system undergoes disintegration when glucose and glucose-containing polymers competitively bind to concanavalin A [18,19]. In addition, glucose-containing insulin [20] or PBA-containing insulin [21] can achieve rhythmic regulation of BGLs by altering the onset and duration of action. Collectively, significant progress has been made in this field; however, the challenge remains to construct a glucose-responsive delivery system that confers good glycemic control and biocompatibility.

Multivesicular liposomes (MVLs) consist of many nonconcentric polyhedral aqueous chambers separated by a network of lipid layers, with a typical particle size ranging from 1 to 100 μm [22,23]. Given their unique structure and large size, MVLs offer the probability of sustained and controlled release of both hydrophilic and lipophilic drugs. Moreover, the structure of the interior compartments can remain intact when exterior compartments are destroyed [24]. Notably, neutral lipids, such as triglycerides, are essential for MVL formation. In MVLs, triglycerides are located in two regions, at bilayer intersection points and in intravesicular triglyceride droplets [25,26]. The release rate of entrapped drugs can be modified by utilizing triglycerides containing alkyl chains of different lengths [24]. Accordingly, MVLs can present several advantages as a drug carrier, including high encapsulation efficiency (EE), high encapsulation volume, good biocompatibility, sustained-release effect, multiple administration methods, and adjustable release rate [27,28,29]. Although MVLs encapsulating insulin have been reported, they are not glucose-responsive and cannot maintain blood glucose homeostasis [23]. If the appropriate glucose-sensitive elements are introduced into MVLs, the resulting delivery system may possess both glucose responsiveness and MVL advantages. However, taking advantage of these glucose-sensitive elements to render MVLs glucose-responsive is challenging.

**Figure 1:**
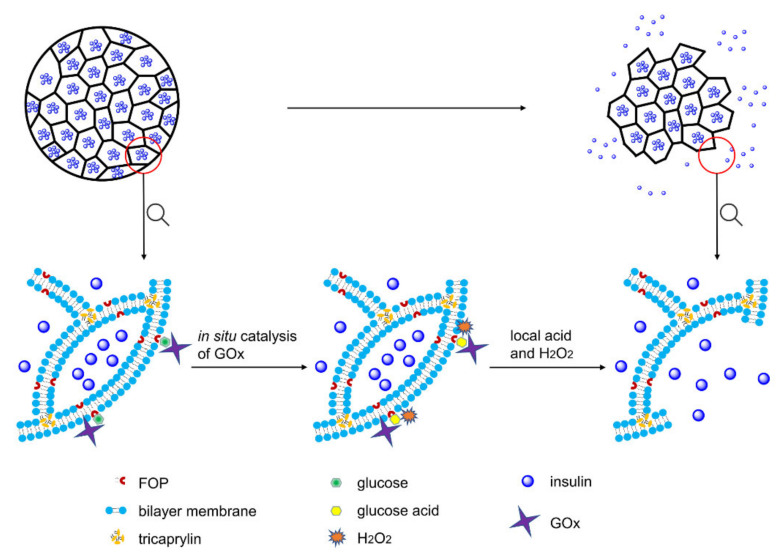
Insulin is released from the glucose-responsive multivesicular liposomes (MVLs) under high glucose conditions. FOP, (3-fluoro-4-((octyloxy)carbonyl)phenyl)boronic acid; GOx, glucose oxidase; H_2_O_2_, hydrogen peroxide.

Herein, we formulated a pH and H_2_O_2_ dual-sensitive MVL using the double emulsion method. The liposomal membrane of MVLs encapsulates (3-fluoro-4-((octyloxy)carbonyl)phenyl)boronic acid (FOP) and 1,2-distearoyl-sn-glycero-3-phosphoethanolamine (DSPE), and the internal vesicles of MVLs encapsulate insulin, GOx, and catalase (CAT). Glucose enrichment on the MVL surface was achieved by the reversible combination of glucose and FOP. As shown in Figure 1, on increasing the local concentration (400 mg/dL), glucose around the MVL membrane undergoes in situ catalyzation by GOx to form gluconic acid and H_2_O_2_, reducing local pH and generating local H_2_O_2_.

Then, DSPE protonation in the liposomal membrane due to pH decrease, as well as lipid peroxidation due to local H_2_O_2_, destroys the outer MVL membrane and, in turn, triggers the release of entrapped insulin. However, the structure of internal vesicles in MVLs remains stable, and the membrane destruction needs to be triggered again prior to insulin release. Under normal glucose conditions (100 mg/dL), the MVL structure can be kept intact, and only a small amount of insulin is released. Furthermore, CAT is beneficial for GOx catalysis as it scavenges H_2_O_2_ and provides oxygen (Figure S1). In the present study, MVL as a drug carrier was used to construct a glucose-responsive system for the first time, and its multivesicular structure affords the basis for glucose-regulated insulin delivery. Moreover, low molecular weight FOP can be easily synthesized with a well-controlled quality; this PBA derivative can be encapsulated into the membrane to achieve glucose enrichment. In addition, the novel trigger mechanism based on in situ catalysis results in glucose-regulated release of insulin. Glucose-responsive MVLs are expected to provide a desirable and intelligent insulin delivery system with rapid response and good biocompatibility, presenting enormous potential in diabetes management.

## 2. Materials and Methods

### 2.1. Materials

Porcine insulin (27 IU/mg) was purchased from Wanbang Biochemical (Xuzhou, China). D(+)-glucose, Alizarin red, glycine, lysine, GOx, and CAT were purchased from Yuanye Biotechnology Co., Ltd. (Shanghai, China). 1,2-dioleoyl-sn-glycero-3-phosphocholine (DOPC), 1,2-dipalmitoyl-sn-glycero-3-phospho-(1′-rac-glycerol) (DPPG), cholesterol, tricaprylin (TC), and DSPE were purchased from Southeast Nano Materials Co., Ltd. (Huai’an, China). Streptozotocin was purchased from BioFroxx (Hesse, Germany). Insulin ELISA kit was purchased from SenBeiJia Biotechnology Co., Ltd. (Nanjing, China). The other chemicals were purchased from Aladdin Chemistry Co., Ltd. (Shanghai, China).

### 2.2. Synthesis and Characterization of PBA Derivatives

**Figure 2:**
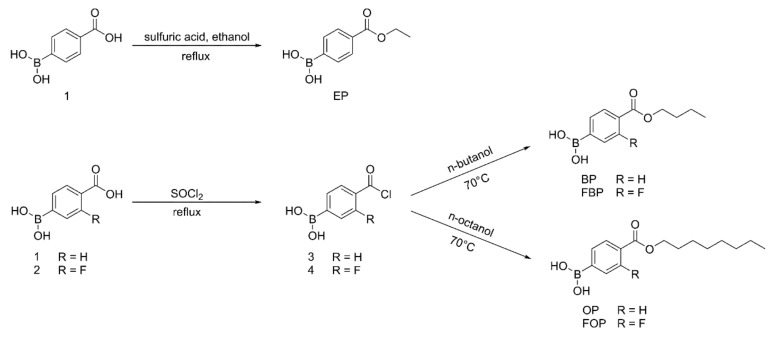
Synthetic routes of compounds EP, BP, FBP, OP, and FOP.

As shown in Figure 2, (4-(ethoxycarbonyl)phenyl)boronic acid (EP) was obtained by refluxing compound 1 with sulfuric acid in absolute ethanol. Briefly, concentrated sulfuric acid (4 mL) was added to a solution of compound 1 (1.28 g, 0.078 mol) in absolute ethanol (160 mL). The reaction mixture was then refluxed for 12 h. After the reaction was completed, the reaction mixture was cooled to room temperature, and ethanol was removed under reduced pressure to obtain the crude product. The crude product was dispersed in water (20 mL). The resulting precipitate was filtered, washed with saturated sodium bicarbonate solution, washed with water, and dried to yield EP.

(4-(butoxycarbonyl)phenyl)boronic acid (BP), (4-(butoxycarbonyl)-3-fluorophenyl)boronic acid (FBP), (4-((octyloxy)carbonyl)phenyl)boronic acid (OP), and FOP were synthesized with modified benzoyl chloride and the corresponding *n*-butanol or n-octanol (Figure 2). Considering FOP as an example, a drop of N, N-dimethylformamide and compound 2 (2.0 g, 0.011 mol) were added to SOCl_2_ (20 mL). The reaction mixture was refluxed for 8 h, and excess SOCl_2_ was removed under reduced pressure to obtain compound 4. Under stirring, compound 4 (1.0 g, 0.005 mol) was added to *n*-octanol (2 mL). The reaction mixture was stirred for 1 h at 70 °C. After completion of the reaction, n-octanol was removed under reduced pressure to obtain an oily liquid. A 10% ethanol solution was added to the oily liquid to precipitate the product. The resulting precipitate was filtered, washed with water, and dried to yield FOP.

The reaction progress was monitored by analytical thin-layer chromatography, and spots were observed under 254 nm ultraviolet (UV) light. The structures of target compounds were determined by high-resolution mass spectrometry (HRMS; Agilent 6520 Q-TOF MS, Santa Clara, CA, USA) and ^1^H nuclear magnetic resonance (^1^H NMR; Bruker AV-300, Bruker, Billerrica, MA, USA). The purity of target compounds was determined by high-performance liquid chromatography (HPLC; LC-20AT, Shimadzu, Kyoto, Japan). The pKa values of PBA derivatives were determined by the titration method.

### 2.3. Preparation of MVLs

**Figure 3:**
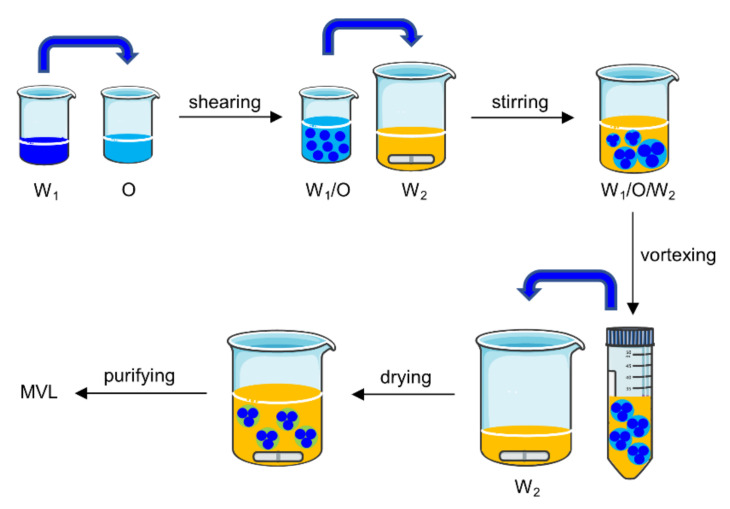
Schematic representation of MVL preparation. W_1_, inter water phase; O, chloroform solution; W_2_, external water phase.

MVLs were prepared using the double emulsion method (Figure 3).

The specific experiments were as follows: (1) 13 mg insulin was dissolved in 1.3 mL HCl solution (pH 2.0). The solution was adjusted to pH 7.0 using an amino acid buffer solution containing 1.2 M glycine and 65 mM lysine; then, 6 mg GOx and 1.5 mg CAT were dissolved in the above solution. The resulting solution was the inner water phase (W_1_). W_1_ was emulsified with 2 mL of a chloroform solution (O), which contained 20 mM neutral phospholipids (containing DOPC and DSPE), 4.2 mM DPPG, 10 mM FOP or FBP, 30 mM cholesterol, and 5 mM TC, by high-speed shearing to obtain a W_1_/O emulsion. To prepare the stable MVLs with pH sensitivity, the molar ratios of DOPC and DSPE were set at 0:1, 1;1, 3:1, and 4:1, respectively. (2) The W_1_/O emulsion was mixed with 16 mL of external water phase (W_2_), which was composed of 306 mM glycine and 1 mM lysine (pH 7.1), by magnetic stirring to obtain a W_1_/O/W_2_ emulsion. (3) The W_1_/O/W_2_ emulsion was further homogenized by vortexing for 30 s in a centrifuge tube. (4) The W_1_/O/W_2_ emulsion was transferred to a beaker containing 16 mL of W_2_; the organic solvent was removed by flushing nitrogen gas over the surface of the mixture at 37 °C at a flow rate of 3 L/min for 20 min. (5) The resulting MVL particles were harvested by centrifugation for 10 min at 600× *g* and washed three times by resuspending in W_2_. The resulting product was resuspended in W_2_ at 20% packed-particle volume per total volume, followed by storage at 2–8 °C for subsequent analysis.

### 2.4. Characterization of MVLs

#### 2.4.1. EEs of Insulin and PBA Derivatives

The EE of insulin is defined as the ratio of the insulin content in MVL particles to the total amount of insulin in the MVL suspension before purification. To determine the EE of insulin, a 0.1 mL of MVL suspension, after nitrogen sweeping, was dissolved by adding 0.5 mL 10% Triton X-100 aqueous solution to detect the total amount of insulin. Next, 0.9 mL of 0.9% saline solution was mixed with another 0.1 mL of MVL suspension. The mixture was centrifuged for 10 min at 600× *g*, and the upper layer of clear liquid was collected to estimate the free insulin. The amount of insulin was detected by HPLC (LC-20AT, Shimadzu, Japan) using an Inertsil ODS-SP column (150 × 4.6 mm, 5 μm; Shimadzu, Kyoto, Japan). The mobile phase consisted of 28% acetonitrile and 72% water (containing 0.2 M Na_2_SO_4_ and 0.46 wt% phosphoric acid; pH was adjusted to 2.3 using triethanolamine), and the flow rate was 1.0 mL/min. The injection volume was 20 μL, and the detection wavelength was 214 nm. The EE was calculated using the following formula: EE (%) = (m_total_ − m_free_)/m_total_ × 100%. Here, m_total_ represents the total amount of insulin and m_free_ represents the free amount of insulin. For determining the EEs of FOP or FBP, 0.9% saline solution was replaced with an amino acid buffer solution containing 133 mM glycine and 150 mM lysine (pH 9.5) as the washing medium; all other steps were the same as for insulin. The leakage rate of insulin was determined using the method used for the EE of insulin.

#### 2.4.2. EEs of GOx and CAT

The method used to determine the EE of GOx was the same as that of insulin. Enzyme activity was used as an index to determine the EE of GOx. The activities of free GOx and total GOx were determined and recorded as U_free_ and U_total_, respectively. The EE of GOx was calculated using the following formula: EE (%) = (U_total_ − U_free_)/U_total_ × 100%. GOx activity was determined using the titration method [30]. Briefly, 1 mL of test solution was added to 25 mL of 60 mM sodium acetate buffer (pH 5.6) containing 2% glucose. The mixture was shaken for 1 h at 30 °C in a water bath with stirring at 200 cycles/min. The reaction was stopped by adding 20 mL of 0.1 M sodium hydroxide solution. The resulting mixture was titrated to a red endpoint using a 0.1 M standard HCl solution using phenolphthalein as an indicator. GOx activity was determined by the volume of the added standard HCl solution. The EE of CAT was determined using the method used for insulin. Enzyme activity was used as an index to determine the EE of CAT. CAT activity was determined using a CAT assay kit in accordance with the manufacturer’s instructions.

#### 2.4.3. Stability of Entrapped Insulin

The stability of entrapped insulin was determined by HPLC and circular dichroism (CD) spectroscopy. In the HPLC method, the insulin solution was prepared according to the preparation method of W_1_ of MVLs. Briefly, insulin was dissolved in HCl solution (pH 2.0) at concentrations of 1, 5, and 10 mg/mL. The solution was adjusted to pH 7.0, with an amino acid buffer solution containing 1.2 M glycine and 65 mM lysine. The resulting solution was incubated at 37 °C in an orbital shaker stirred at 12 cycles/min. The insulin content of the incubation solution was determined by HPLC at each set time point and compared with that at 0 h.

The secondary structural change in insulin released from MVLs was evaluated as reported previously [31]. The insulin solution (130 μg/mL) was used as the standard CD spectrum. The MVL suspension stored at 2–8 °C for 1 month was diluted in release media, and the mixture was incubated at 37 °C in a water bath. The released insulin was obtained by centrifugation, and the insulin concentration was adjusted to 130 μg/mL for CD measurement. Test solution spectra were recorded in the range of 200–250 nm at 25 °C using a CD spectrometer (J-800, JASCO, Tokyo, Japan).

#### 2.4.4. Morphology and Particle Size of MVLs

The MVL morphology was examined using both optical microscopy (BX-53, Olympus, Tokyo, Japan) and cryogenic-scanning electron microscopy (cryo-SEM; Quanta 450, FEI, Hillsboro, OR, USA). The cross-section of MVLs could be observed by cryo-SEM. Briefly, conductive carbon adhesive was put on the sample table, and the diluted MVLs were placed on the conductive carbon adhesive. Then, the sample table with MVLs was rapidly frozen in liquid nitrogen for 30 s and transferred to the preparation chamber in a vacuum state for sublimation gold-coating. Finally, the sample was fractured and an approximately 5 nm of gold layer was sputter-coated on the exposed surface. The sample was imaged in the pre-cooled (~ −140) FEI Quanta450 SEM (FEI, Hillsboro, OR, USA) operated at 5 kV. The particle size distribution and average particle size of the MVLs were measured using a laser particle size analyzer (Bettersize2600, Better, Dandong, China).

#### 2.4.5. Fourier Transform Infrared (FTIR) Analysis

The FTIR spectra of FOP, liposomal membranes without FOP, and liposomal membranes with FOP were recorded using an FTIR spectrometer (Tensor 27, Bruker, Ettlingen, Germany). The liposomal membranes were prepared as follows: The membrane materials were dissolved in chloroform and dried under evaporation in a rotary evaporator (RV-10, IKA, Staufen, Germany) at 35 °C. The three samples were compressed in a potassium bromide pellet and scanned for spectra in the range of 4000–400 cm^−1^ at a resolution of 1 cm^−1^.

#### 2.4.6. Differential Scanning Calorimetry (DSC) Analysis

The thermal properties of FOP, liposomal membranes without FOP, and liposomal membranes with FOP were investigated by DSC (DSC 3500, Netzsch, Selb, Germany). Liposomal membranes were prepared in the same way as described in Section 2.4.5. The three samples were weighed and measured in a calorimeter pool. The temperature range was −30 to 200 °C, and the temperature rise rate was 10 °C/min.

### 2.5. In Vitro Insulin Release from MVLs

Briefly, 10 mM phosphate-buffered saline (PBS; pH 7.4) with different glucose concentrations (0, 100, or 400 mg/dL) was used to perform the in vitro drug release experiments. Aliquots of MVL suspensions were added to different Eppendorf tubes containing the release media. The mixture was incubated at 37 °C in a water bath stirred at 12 cycles/min. Additionally, insulin release from MVLs was assessed by changing the glucose concentration from 100 to 400 mg/dL in PBS (pH 7.4). The MVL particles were first incubated in PBS (pH 7.4) with 100 mg/dL glucose for 10 h, followed by the addition of glucose to achieve a concentration of 400 mg/dL. At indicated time points, tubes (three tubes per time point) were centrifuged at 600× *g* for 5 min after 3-fold PBS (pH 7.4) was added. The supernatant solution was removed. MVL particles were lysed with 10% Triton X-100 aqueous solution, and the amount of insulin in the MVL particles was detected by HPLC. The release rate was calculated using the following formula: release rate (%) = (m_total_ − m_particle_)/m_total_ × 100%. Here, m_particle_ represents the amount of insulin in MVL particles and m_total_ represents the total amount of insulin in the same amount of MVL suspension. Meanwhile, the average particle size of MVLs was measured during the release process using a laser particle size analyzer. The glucose-dependent pulsatile test was conducted to further access the glucose responsiveness of the MVLs. First, MVL particles were incubated in 100 mg/dL glucose solution for 2 h. The supernatant solution was removed after MVL particles were centrifuged, and the insulin concentration in the supernatant solution was detected by HPLC. The MVL particles were resuspended in 400 mg/dL glucose solution for another 2 h. The cycles were repeated three times.

### 2.6. Interaction between 1,2—Diols and FOP

Alizarin red with an ortho-hydroxyl group was selected as the probe to examine the interaction between 1,2-diols and FOP. UV absorption spectra of 10^−4^ M Alizarin red (a), a mixture of 10^−4^ M Alizarin red and 10^−3^ M FOP (b), and a mixture of 10^−4^ M Alizarin red, 10^−3^ M FOP, and 10^−1^ M glucose (c) in PBS (pH 7.4) were determined in the range of 390–600 nm using a UV spectrophotometer (UV-2600, Shimadzu, Kyoto, Japan). Ethanol was added to the PBS (pH 7.4) to improve FOP solubility in the solvent. The color of each solution was also determined.

### 2.7. Interaction between Alizarin Red and MVL Membrane

An appropriate amount of freshly prepared Alizarin red solution was added to MVL encapsulating enzymes and insulin (MVL(E+I)), or MVL encapsulating FOP, enzymes, and insulin (MVL(F+E+I)) suspensions, respectively, and the samples were incubated at 37 °C in a water bath. At indicated time points, MVL particles were washed with PBS (pH 7.4) and observed under an optical microscope. The morphology and color of the MVL particles were examined to analyze the interaction between Alizarin red and the MVL membrane.

### 2.8. pH and H_2_O_2_ Sensitivities of MVLs

MVLs were incubated in PBS with different concentrations of gluconic acid or H_2_O_2_ to assess the pH or H_2_O_2_ sensitivities of MVLs, respectively. The concentrations of gluconic acid and H_2_O_2_ in the release media were determined according to the BGLs under physiological conditions [32,33]. The release media containing gluconic acid at different pH values (pH = 7.4, 6.3, 5.2, or 4.0) and the release media with different concentrations of H_2_O_2_ (0, 5, 10, or 25 mmol/L) were used for the in vitro experiments. The in vitro release method was the same as that described in Section 2.5.

### 2.9. Interaction between GOx and MVL mMembrane

#### 2.9.1. Fluorescence Spectroscopy

In brief, GOx was dissolved in PBS (pH 7.4) at a concentration of 4 mg/mL, and the fluorescence emission spectra of the GOx solutions, both in the absence and presence of blank MVLs, were collected using a fluorescence spectrometer (RF-5301PC, Shimadzu, Kyoto, Japan). Fluorescence emission spectra (excitation at 282 nm) were recorded in the range of 305–420 nm at 25 °C, with an excitation slit width of 20 nm and an emission slit width of 5 nm. The maximum emission wavelength of each spectrum was recorded, and the blue or red shift of the maximum emission wavelength reflected the interaction of GOx and the MVL membrane [34].

#### 2.9.2. Docking Study

The X-ray crystallographic structure of GOx (PDB code: 3QVR) was obtained from the Protein Data Bank. DSPE, DOPC, DPPG, FOP, and cholesterol were preprocessed using ChemBio3D Ultra 14.0 (PerkinElmer, Waltham, MA, USA) and ChemBioDraw Ultra 14.0 (PerkinElmer, Waltham, MA, USA). The interactions between GOx and membrane materials were performed using AutoDock Tools 1.5.6 (Scripps Research Institute, San Diego, CA, USA) and AutoDock Vina (Scripps Research Institute, San Diego, CA, USA). The docking results were analyzed using PyMOL (Schrodinger, New York, NY, USA) [35]. Hydrogen bonds and docking diagram between GOx and membrane materials were recorded to analyze the interaction between GOx and the MVL membrane.

### 2.10. In Vivo Studies for Type 1 Diabetes Treatment

The diabetic rat (male Sprague-Dawley rat) model was established by streptozotocin administration according to a previously described method [36,37]. Fasting BGLs were measured using a blood glucose meter (5DM-2A, Yicheng Biotech. Co., Ltd., Beijing, China). Hyperglycemic rats that maintained stable BGLs (> 16.7 mmol/L) were randomly divided into four groups (five rats per group) and subcutaneously administered MVL(E+I), MVL(F+E+I), MVL encapsulating FOP and insulin (MVL(F+I)), and MVL encapsulating FOP and enzymes (MVL(F+E)), respectively. The insulin dosage was set at 12 IU/kg for MVL treatments. BGLs were measured using a blood glucose meter at specific time points after administration. Meanwhile, the concentration of serum insulin was determined using an insulin ELISA kit in accordance with the manufacturer’s instructions. Moreover, to perform an intraperitoneal glucose tolerance test (IPGTT), diabetic rats were administered a glucose injection (2 g/kg). Hyperglycemic rats were fasted for 6 h, with free access to water before IPGTT. A single IPGTT was performed 3 h after administering MVL(F+E+I) or insulin solution. The insulin dosage was set at 5 IU/kg for MVL(F+E+I) and the insulin solution. Multiple IPGTTs were performed 2 h post-MVL(F+E+I) administration, and the insulin dosage was set at 12 IU/kg. Glucose solution was injected every 2 h, thrice in total. Multiple IPGTTs in healthy rats were used as controls. In addition, the side effects were evaluated in healthy rats by administering MVL(F+E+I) and insulin solution.

### 2.11. Cytotoxicity Study

The cytotoxicity of PBA derivatives was examined in HeLa cells using the 3-(4,5)-dimethylthiahiazo(-z-yl)-3,5-di-phenytetrazoliumromide (MTT) assay [38]. Briefly, HeLa cells were seeded in 96-well plates at a density of 6000 cells per well and cultured for 24 h. Then, serially diluted PBA derivatives (ranging from 0.001 to 10 μg/mL) were added to the wells. After 24 h of incubation at 37 °C, the cells were subjected to the MTT assay.

### 2.12. Histopathological Evaluation

Briefly, diabetic rats subcutaneously administered MVL(F+E+I) or the blank solution (306 mM glycine and 1 mM lysine; pH 7.1) were euthanized by CO_2_ asphyxiation; the insulin dosage of MVL(F+E+I) was set at 12 IU/kg. Then, 24 h after injection, the tissues around the injection site were excised, fixed in 10% formalin, embedded in paraffin, cut into 5 μm sections, and stained with hematoxylin and eosin (H&E) for histological analysis.

### 2.13. Statistical Analysis

All data are presented as mean ± standard deviation (SD). Statistical analysis was performed using one-way ANOVA or unpaired *t*-test, and *p* < 0.05 was deemed a minimal level of significance.

## 3. Results

### 3.1. Synthesis and Characterization of PBA Derivatives

Herein, we attempted to obtain membrane materials with a high affinity to glucose. Accordingly, a series of PBA derivatives were designed and synthesized by modifying 4-borono-2-fluorobenzoic acid or 4-boronobenzoic acid with different types of fatty alcohols (Figure 2). The related esterification and acylation possess advantages of minimal time requirement and high yield. The purity of target compounds was > 97%. As shown in Section 1 and Section 2 (Figures S2–S10) in the Supplementary Materials, the target compound structures were confirmed by HRMS and ^1^H NMR. Among these compounds, FBP, OP, and FOP were the first to be reported. According to the mass spectra of these PBA derivatives, multiple molecular ion peaks were detected, as naturally occurring boron contains two types of stable isotopes. In terms of the hydrogen spectra of these PBA derivatives, the chemical shift of benzene ring hydrogens appeared at 7.5–8.5 ppm, and the chemical shift of alkyl hydrogens appeared at 0.9–2.0 ppm.

To evaluate the binding affinity between PBA derivatives and glucose, the pKa values of PBA derivatives were determined experimentally. The pKa values of EP, BP, FBP, OP, and FOP were 7.56, 7.53, 6.79, 7.84, and 6.90, respectively. PBA derivatives with lower pKa values have a higher affinity for 1,2- or 1,3-diols and are more suitable for participating in the construction of glucose-responsive systems [39]. Therefore, FBP and FOP were selected for further studies.

### 3.2. Preparation of MVLs

We obtained the optimal formulation by screening membrane materials of MVLs. The molar ratio of neutral phospholipids, DPPG, cholesterol, and TC in the formulation was established based on previous reports [23,40], which could ensure the stability of the resulting MVLs. Then, the molar ratio of DOPC to DSPE was further screened when the total number of moles of neutral phospholipids was kept constant. As listed in Table 1, when the molar ratio of DOPC to DSPE was 0:1, the MVLs could not be prepared.

When the molar ratio of DOPC to DSPE was 4:1, the EE of insulin was 63.08 ± 2.92%. Our findings indicated that the EE of insulin increased with an increase in the DOPC ratio. F3 and F4, with high EEs, were selected to investigate the pH sensitivity of MVL particles. The pH sensitivity of MVLs was investigated by observing the morphology of MVLs incubated in PBS (pH 4.0). The pH value of PBS for screening was set at 4.0, in accordance with the pH value associated with elevated glucose concentrations, which will be further explained in a later subsection. The structure of F3 was destroyed, whereas that of F4 remained intact (Table 1). Accordingly, the lower the molar ratio of DOPC, the stronger the pH sensitivity and lower the EE of insulin. Therefore, F3 was selected to further screen the MVL formulation.

Next, by utilizing FBP or FOP as membrane materials, the EEs of insulin and PBA derivatives were investigated. No significant difference in the EE of insulin was observed between the two formulations. However, the EE of FBP (3.10 ± 0.52%) was considerably lower than that of FOP (18.75 ± 2.50%). Thus, FOP was deemed more suitable than FBP for participating in the construction of lipid membranes. The optimal formulation was determined using the above experiments.

### 3.3. Characterization of MVLs

#### 3.3.1. EEs of MVL Contents

For MVL(F+E+I), the EEs of insulin, GOx, and CAT were 49.79 ± 1.50%, 43.48 ± 2.95%, and 45.68 ± 3.12%, respectively. Next, MVLs with different formulations were prepared based on the optimal formulation. For MVL(E+I) and MVL(F+I), the EEs of insulin were 44.16 ± 2.22% and 55.21 ± 2.57%, respectively. The results revealed that the addition of FOP and enzymes had no pronounced effect on the EE of insulin. On washing the MVLs to remove free insulin, we found that almost all the insulin was encapsulated in the interior compartments of final MVLs. The insulin leakage rate from MVL(F+E+I) was < 5% and remained almost constant in a month, indicating that MVL(F+E+I) remained stable upon storage at 2–8 °C (Table S1).

#### 3.3.2. Stability of Entrapped Insulin

**Figure 4:**
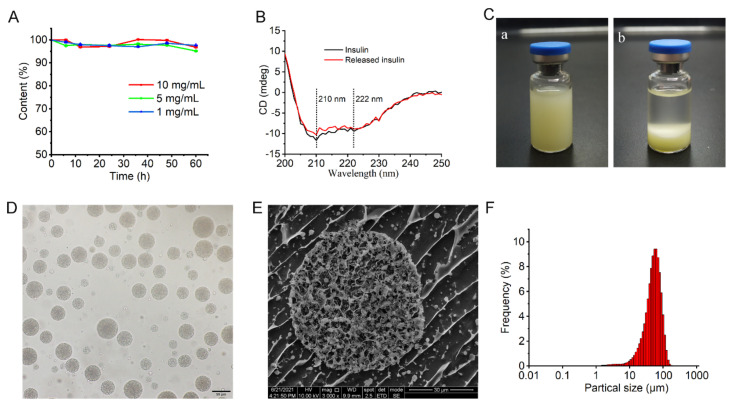
Characterization of MVL(F+E+I): (**A**) Stability of insulin solutions at different concentrations. Data are shown as mean ± SD (*n* = 3). (**B**) CD spectra of insulin and insulin released from MVLs. (**C**) Appearance of MVL(F+E+I) suspension (**a**); appearance of MVL(F+E+I) suspension after standing for 1 d at 4 °C (**b**). (**D**) Photomicrograph of MVL(F+E+I). Scale bar = 50 μm. (**E**) Cross-sectional view of MVL(F+E+I) observed by cryo-SEM. Scale bar = 30 μm. (**F**) Size distribution of MVL(F+E+I) determined using a laser particle size analyzer.

According to Section 2.3, the insulin concentration in W_1_ of the MVLs was 10 mg/mL. We assessed the stability of insulin at different concentrations (1, 5, and 10 mg/mL) by HPLC as the insulin concentration in the MVLs gradually decreased during the release period. As shown in Figure 4A, the insulin content in the incubation solution remained constant for 60 h at 37 °C at insulin concentrations of 1, 5, and 10 mg/mL, respectively.

The results indicated that insulin remained stable in the MVLs during the release period. Notably, the secondary structure of insulin is fundamental for its biological activity and plays a vital role in the interaction between insulin and its receptor [31]. As shown in Figure 4B, we detected two negative bands for released insulin at 210 nm and 222 nm. The bands at 210 and 222 nm correspond to the α-helix and β-pleated sheet structures, respectively. No differences in the shape and degree of ellipticity were observed between fresh insulin and insulin released from MVLs; this indicated that the secondary structure of insulin released from MVLs was unaltered. Accordingly, the entrapped insulin was stable during the preparation, storage, and release processes.

#### 3.3.3. Appearance, Morphology, and Particle Size of MVLs

The GOx solution appeared pale yellow in color. The MVL(F+E+I) suspension also appeared pale yellow owing to the entrapped GOx (Figure 4Ca). The micron-sized MVL(F+E+I) particles gradually settled to the bottom (Figure 4Cb). Under a light microscope, the MVL(F+E+I) particles were smooth and spherical (Figure 4D). As shown in Figure 4E, the cross section of MVL(F+E+I) was roughly circular, and the nonconcentric internal chambers of MVL(F+E+I) were observed. Figure 4F shows the representative particle size distribution of MVL(F+E+I), with a volume-weighted median diameter of 55.81 μm, and 90% of the particles were sized between 10 and 100 μm. Thus, the prepared MVL(F+E+I) was stable in quality and suitable for subcutaneous injection based on its micron size.

#### 3.3.4. FTIR and DSC Analysis

**Figure 5:**
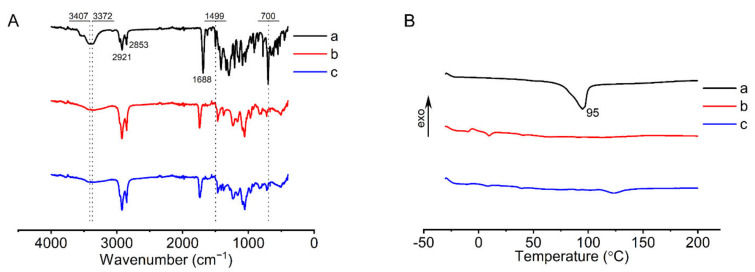
Characterization of MVL(F+E+I): (**A**) FTIR spectra of FOP (**a**), liposomal membranes without FOP (**b**), and liposomal membranes with FOP (**c**). (**B**) DSC profiles of FOP (**a**), liposomal membranes without FOP (**b**), and liposomal membranes with FOP (**c**).

The FTIR and DSC analysis were performed to determine FOP distribution in the liposomal membrane [41]. In the FTIR spectrum of FOP (Figure 5A), the peaks at 3407 cm^−1^ and 3372 cm^−1^ were ascribed to the O–H stretching vibration, while those at 1499 cm^−1^ and 700 cm^−1^ were ascribed to the C=C stretching vibration and C–H bending vibration of the benzene ring, respectively.

However, these characteristic peaks could not be observed in the FTIR spectra of liposomal membranes. As shown in Figure 5B, an endothermic peak appeared at 95 °C in the DSC thermogram of FOP, and this peak represented the melting point of FOP. In contrast, the DSC thermograms of liposomal membranes showed no peaks at 95 °C. Collectively, the characteristic peaks of FOP in liposomal membranes disappeared in the FTIR spectra and DSC thermograms, suggesting that FOP was uniformly distributed in the liposomal membrane in an amorphous or molecular state.

### 3.4. In Vitro Insulin Release from MVLs

**Figure 6:**
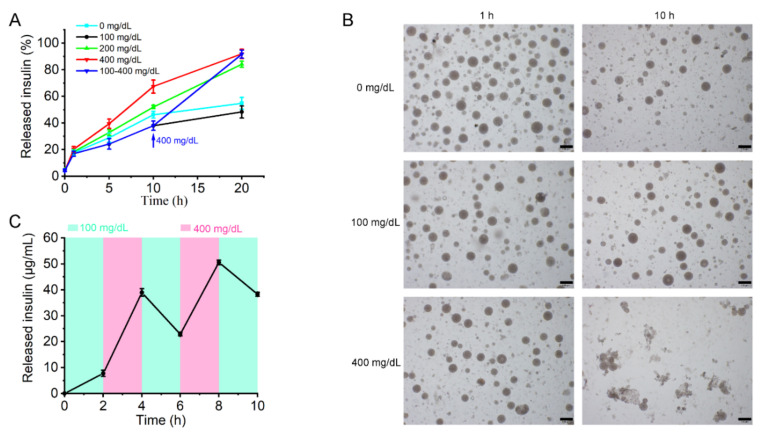
Insulin release of MVL(F+E+I) in vitro: (**A**) In vitro accumulated insulin release from MVL(F+E+I) in PBS (pH 7.4) with different glucose concentrations. Data are shown as mean ± SD (*n* = 3). (**B**) Glucose concentration-dependent morphology changes in MVL(F+E+I) in PBS (pH 7.4) at 1 and 10 h. Scale bar = 100 μm. (**C**) Pulsatile release profile of MVL(F+E+I) presents the rate of insulin release as a function of glucose concentrations. Data are shown as mean ± SD (*n* = 3).

The glucose responsiveness of the MVLs was investigated using in vitro release tests. As shown in Figure 6A, MVL(F+E+I) in 100 mg/dL and 400 mg/dL glucose solutions released 29% and 39% insulin in 5 h and 48% and 92% insulin in 20 h, respectively.

In the glucose-free or 100 mg/dL glucose solutions, the particle number was marginally reduced, with the structure of most MVL(F+E+I) particles intact for 10 h. However, almost no intact structures were observed in the 400 mg/dL glucose solution at 10 h (Figure 6B). The particle size of MVL(F+E+I) decreased gradually over time in the 100 and 400 mg/dL glucose solutions. Notably, the particle size of MVL(F+E+I) decreased quickly in the 400 mg/dL glucose solution when compared to the 100 mg/dL solution (Figure S11). In addition, from 10 to 20 h, the insulin release rate from MVL(F+E+I) was increased when the glucose concentration of the incubation solution was altered from 100 mg/dL to 400 mg/dL (Figure 6A). These results revealed that MVL(F+E+I) could control insulin release based on glucose concentrations in the environment. For MVL(E+I), no significant difference in release behavior was observed in 100 and 400 mg/dL glucose solutions (Figure S12). In addition, MVL(E+I) demonstrated a higher burst release than MVL(F+E+I). FOP was found to increase the stability of MVLs, while decreasing burst release.

For MVL(F+E+I), pulsatile insulin release was achieved by altering glucose concentrations in the release media (Figure 6C). During the initial 2 h, the insulin concentration was 7.75 μg/mL in the 100 mg/dL glucose solution. Then, at 4 h, the insulin concentration increased to 38.97 μg/mL in the 400 mg/dL glucose solution. At 2, 6, and 10 h, the insulin concentration in the 100 mg/dL glucose solution was relatively low. At 4 h and 8 h, the insulin concentration in the 400 mg/dL glucose solution was relatively high. The amount of insulin release was altered based on fluctuating glucose concentrations in the release media. Collectively, the in vitro release experiments substantiated that the release of insulin from MVL(F+E+I) underwent a glucose-mediated process.

### 3.5. Glucose-Responsive Mechanism of MVLs

#### 3.5.1. Glucose Enrichment for MVL(F+E+I)

**Figure 7:**
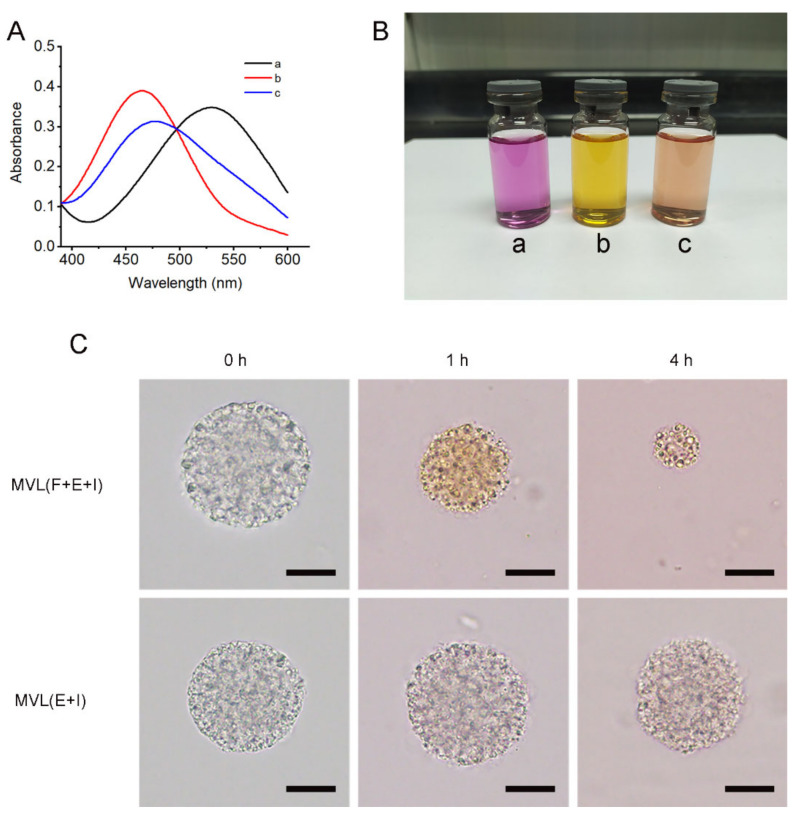
Glucose-responsive mechanism of MVL(F+E+I): (**A**) UV absorption spectra of 10^−4^ M Alizarin red (**a**), a mixture of 10^−4^ M Alizarin red and 10^−3^ M FOP (**b**), and a mixture of 10^−4^ M Alizarin red, 10^−3^ M FOP and 10^−1^ M glucose (**c**) in PBS (pH 7.4). (**B**) Colors of solutions a, b, and c in Figure 6A. (**C**) Photomicrographs of MVL(F+E+I) and MVL(E+I) in PBS (pH 7.4) with 1% Alizarin red at 0 h, 1 h, and 4 h, respectively. Scale bar = 20 μm.

We attempted to select an appropriate probe to examine the role of FOP in MVLs. As shown in Figure 7A, a shift in UV λmax from 529 nm of solution a to 465 nm of solution b was observed when FOP was added, suggesting that FOP boric acid interacted with Alizarin red hydroxyl.

A shift in UV λmax from 465 nm of solution b to 477 nm of solution c was detected when glucose was added; this indicated that glucose competed with Alizarin red to bind with FOP through reversible boronate formation. In addition, the colors of the test solutions were altered with UV λmax. As shown in Figure 7B, the colors of solutions a, b, and c were pale red, yellow, and pale orange, respectively. The results indicated that the binding of Alizarin red to FOP altered the visible light absorption wavelength. Thus, the Alizarin red was used in the following experiments based on these results.

MVL(F+E+I) or MVL(E+I) was incubated in the Alizarin red solution, and the changes in MVL particles are shown in Figure 7C. Following incubation for 1 h, the particle size of MVL(F+E+I) was reduced and the particle color changed from gray to yellow. In the case of MVL(E+I), the particle size mainly remained unaltered and the particle color changed from gray to light red. These results indicated that the FOP of MVL(F+E+I) could bind with Alizarin red, resulting in a change in particle color; the altered solution color was consistent with the results of Figure 7B. MVL(E+I) particles appeared pale red due to the diffusion of Alizarin red. On incubating MVLs for 4 h, the particle size of MVL(F+E+I) continued to decrease, whereas the particle size of MVL(E+I) mainly remained unaltered. The local acidic environment was formed by enriching Alizarin red on the surface of MVL(F+E+I), with the outer structure of MVL(F+E+I) then destroyed. Accordingly, we concluded that 1,2-diols, including glucose, could accumulate around the MVL(F+E+I) membrane, and the structure of MVL(F+E+I) would be further destroyed by the local acidic environment induced by gluconic acid production.

#### 3.5.2. pH and H_2_O_2_ Sensitivities of MVL(F+E+I)

**Figure 8:**
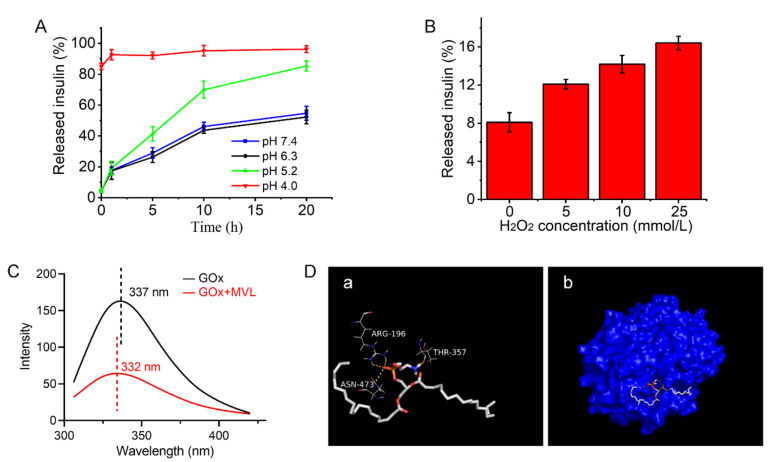
Glucose-responsive mechanism of MVL(F+E+I): (**A**) In vitro accumulated insulin release from MVL(F+E+I) in PBS with different gluconic acid concentrations at different pH levels. Data are shown as mean ± SD (*n* = 3). (**B**) In vitro accumulated insulin release from MVL(F+E+I) in PBS (pH 7.4) with different H_2_O_2_ concentrations at 0.5 h. The H_2_O_2_ concentrations in the release media were calculated according to the BGLs under physiological conditions. Data are shown as mean ± SD (*n* = 3). (**C**) Fluorescence emission spectra of GOx solutions in the presence and absence of blank MVLs. (**D**) Docking study of the interaction of DSPE with GOx. DSPE is shown as sticks. (**a**) Residues involved in the interaction with DSPE are labeled and shown as lines, with the remainder of GOx not shown. Hydrogen bonds are indicated by yellow doted lines. (**b**) GOx is shown as a surface.

We performed in vitro release tests to further investigate the glucose-responsive mechanism of MVL(F+E+I). Following the conversion of glucose (0, 100, 200, and 400 mg/dL) in the solution to gluconic acid and H_2_O_2_ under GOx catalysis, the corresponding concentrations of gluconic acid and H_2_O_2_ could be calculated. The concentrations of gluconic acid and H_2_O_2_ in the release media were established based on the calculated concentrations. Therefore, glucose concentrations of 0, 100, 200, and 400 mg/dL corresponded with pH values of 7.4, 6.3, 5.2, and 4.0, respectively [32]. In addition, glucose concentrations of 0, 100, 200, and 400 mg/dL corresponded with 0, 5, 10, and 25 mmol/L H_2_O_2_, respectively [33]. As shown in Figure 8A, rapid insulin release was achieved at pH 5.2 and 4.0.

However, only a small amount of insulin was released from MVL(F+E+I) at pH 7.4 and 6.3. These results indicated that low pH accelerated insulin release. As presented in Figure 8B, the higher the H_2_O_2_ concentrations, the higher the insulin release rate. The corresponding pH and H_2_O_2_ concentration in the 400 mg/dL glucose solution could induce rapid insulin release; however, insulin release in the 100 mg/dL glucose solution was gradual. Collectively, MVL(F+E+I) modulated insulin release in response to glucose by varying hydrogen ion and H_2_O_2_ concentrations.

#### 3.5.3. Interaction between GOx and MVL Membrane

The interaction between GOx and the MVL membrane also mediates the glucose responsiveness of MVLs. As shown in Figure 8C, the GOx solution showed a maximum emission wavelength of 337 nm. The GOx solution in the presence of blank MVLs exhibited a maximum emission wavelength of 332 nm. The addition of blank MVLs resulted in a modest blue shift of 5 nm. The 5 nm shift in the emission maximum indicated that some amino acid residues of GOx moved from the hydrophilic environment to the hydrophobic environment, suggesting that GOx inserted into the MVL membrane [34].

As shown in Figure 8Da, the oxygen atom of the DSPE phosphate group formed hydrogen bonds with the amino acid residues ARG-196 and ASN-473, respectively. The amino group of DSPE formed a hydrogen bond with the amino acid residue THR-357. As shown in Figure 8Db, the hydrophilic end of DSPE formed multiple hydrogen bonds with amino acid residues on the GOx surface, and the hydrophobic end of DSPE was located outside the protein, which indicated that the MVLs, including DSPE, formed strong hydrogen bonds with GOx based on the DSPE distribution in the bilayer lipid membrane. Although DOPC, DPPG, and FOP could also form hydrogen bonds with GOx, these small molecular compounds were located inside the protein (Figures S13–S15). DOPC, DPPG, and FOP in the lipid membrane could only form weak hydrogen bonds with GOx. In addition, no interaction was observed between cholesterol and GOx (Figure S16). In conclusion, the interactions between GOx and MVLs were confirmed by fluorescence experiments and molecular docking.

### 3.6. In Vivo Studies for type 1 Diabetes Treatment

**Figure 9:**
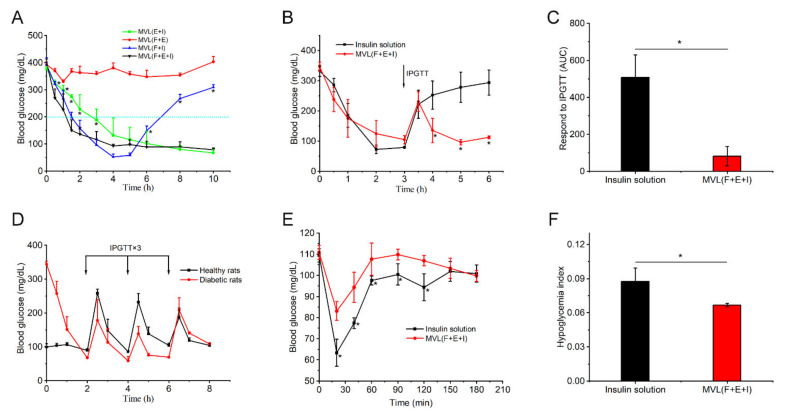
In vivo glucose regulation of MVLs on streptozotocin-induced diabetic rats: (**A**) BGLs of streptozotocin-induced diabetic rats subcutaneously administered MVL(E+I), MVL(F+E), MVL(F+I), and MVL(F+E+I). Statistical significance was determined by one-way ANOVA (* *p* < 0.05 for administration with MVL(F+E+I) compared with MVL(E+I) or MVL(F+I)). (**B**) Single IPGTT toward diabetic rats treated with MVL(F+E+I) and insulin solution. Statistical significance was evaluated by a two-tailed, unpaired *t*-test (* *p* < 0.05). (**C**) Responsiveness was calculated based on the area under the curve (AUC) from 3 to 6 h, with the baseline set at the 3 h blood glucose reading. Statistical significance was evaluated by a two-tailed, unpaired *t*-test (* *p* < 0.05). (**D**) Multiple IPGTTs toward diabetic rats treated with MVL(F+E+I) in comparison to the healthy rats. (**E**) Blood glucose changes in healthy rats treated with MVL(F+E+I) and insulin solution. Statistical significance was evaluated by a two-tailed, unpaired *t*-test (* *p* < 0.05). (**F**) Quantification of hypoglycemia index of MVL(F+E+I) and insulin solution. Statistical significance was evaluated by a two-tailed, unpaired *t*-test (* *p* < 0.05). Data are shown as mean ± SD (*n* = 5).

The in vivo hypoglycemic effect of MVLs was evaluated in diabetic rats. As shown in Figure 9A, MVL(F+E+I) rapidly reduced BGLs and maintained levels within the normal range without inducing the risk of hypoglycemia.

However, MVL(F+E), MVL(E+I), and MVL(F+I) failed to achieve an adequate hypoglycemic effect. MVL(F+E) did not reduce BGLs owing to the lack of insulin. Correspondingly, serum insulin levels in rats treated with MVL(F+E+I) were consistent with BGLs (Figure S17). The serum insulin levels of diabetic rats were relatively high under hyperglycemic conditions, whereas those were relatively low under normoglycemic conditions. IPGTT was performed to further investigate the in vivo hypoglycemic effect of MVL(F+E+I). For single IPGTT, diabetic rats that received MVL(F+E+I) showed a spike in BGLs after glucose stimulation; this was followed by a rapid decrease to normoglycemic levels in 2 h (Figure 9B). Although the diabetic rats treated with MVL(F+E+I) underwent glucose stimulation, the BGLs could be regulated within the normal range. In diabetic rats administered the insulin solution, the BGLs were rapidly increased and maintained at hyperglycemic levels after the intraperitoneal glucose injection (Figure 9B); this was due to the rapid metabolism of insulin. The diabetic rats treated with MVL(F+E+I) showed significantly enhanced glucose tolerance following the glucose challenge when compared with those treated with insulin solution (Figure 9C). For multiple IPGTTs, the blood glucose fluctuations in diabetic rats treated with MVL(F+E+I) were similar to those observed in healthy rats after multiple glucose stimulations (Figure 9D). During the experimental period, the BGLs of diabetic rats were maintained within the normal range (< 200 mg/dL), suggesting that MVL(F+E+I) could effectively reduce BGLs without inducing a significant fluctuation.

We next assessed the risk of hypoglycemia associated with treatment by MVL(F+E+I) and insulin solution. As shown in Figure 9E, the BGLs of insulin-treated healthy rats were markedly reduced, whereas the BGLs of healthy rats treated with MVL(F+E+I) showed a marginal reduction. The corresponding hypoglycemia index is defined as the fall in glucose from the initial reading to the nadir divided by the time over which this fall is reached. A significantly lower hypoglycemic index was recorded in the MVL(F+E+I)-treated group than in the insulin-treated group (Figure 9F). Consequently, MVL(F+E+I) confers a rapid, intelligent, and safe hypoglycemic effect for diabetes treatment.

### 3.7. Biocompatibility Study

**Figure 10:**
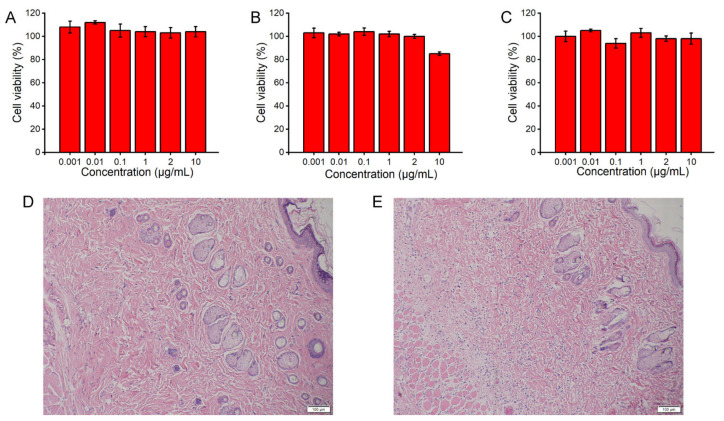
Biocompatibility study of MVL(F+E+I): Cytotoxicity assays of FBP (**A**), OP (**B**), and FOP (**C**) toward HeLa cells for 24 h. Data are shown as mean ± SD (*n* = 6). H&E stained sections of subcutaneously injected blank solution (**D**) or MVL(F+E+I) (**E**) with surrounding tissue after 24 h, respectively. Scale bar = 100 μm.

Based on the structure of PBA derivatives, FBP, OP, and FOP were selected for cytotoxicity assessment using the MTT assay. As shown in Figure 10A–C, the PBA derivatives showed no significant cytotoxicity in HeLa cells and could be used to construct a glucose-responsive insulin delivery system. 

As shown in Figure 10D,E, almost no visible inflammation was observed in diabetic rats with MVL(F+E+I) treatment when compared with the blank solution. The transient acidic and H_2_O_2_-enriched environment induced no adverse reactions around the injection site. In conclusion, MVL(F+E+I), mainly composed of phospholipids and cholesterol, is non-toxic and suitable for insulin delivery.

## 4. Discussion

In patients with diabetes, the large fluctuation in BGLs has been associated with several complications, and the use of glucose-responsive insulin delivery systems is an important means to maintain blood glucose homeostasis. Previous studies examining potential glucose-responsive systems have largely focused on nanoparticles, microgels, and micelles [42]; however, it is difficult to achieve good glucose control and biocompatibility for these delivery systems. In the present study, glucose-sensitive elements were introduced into MVLs to render formulated MVLs glucose-responsive. In the MVL preparation process (Figure 3), W_2_ was added twice. An appropriate volume of W_2_ was added the first time to efficiently form a W_1_/O/W_2_ emulsion; it was then added a second time to accelerate the removal of the organic solvent. After the formation of the W_1_/O/W_2_ emulsion, further vortexing allowed the formation of uniformly sized MVL particles. In addition, W_1_ and W_2_ should have similar pH values, which is critical for preparing pH-sensitive MVLs. If the pH value of W_1_ significantly differs from that of W_2_, the flow of hydrogen ions between W_1_ and W_2_ can cause structural changes in the DSPE, decreasing the stability of the MVLs [43]. The EE of insulin increased with a decrease in the DSPE ratio (Table 1); this could be attributed to the rigidity of DSPE. The alkyl chain of DOPC contains two double bonds, whereas that of DSPE is saturated. On decreasing the DSPE ratio, the rigidity of liposomal membranes decreases to stabilize the structure of MVLs, thus increasing the EE of insulin. For the final MVLs, almost all the insulin was encapsulated in the internal vesicles; this affords the basis for sustained and controlled insulin release [23]. Insulin encapsulated in the MVLs accumulates at the injection site, and released insulin is absorbed into the blood and exerts its pharmacological effect.

Given its large size, MVL(F+E+I) remains at the injection site to slowly release insulin following subcutaneous administration. Cryo-SEM image indicates that the multivesicular structure of MVL(F+E+I) was slightly destroyed (Figure 4E); this was because ice crystals or mechanical forces led to the destruction of liposomal membranes during sample manipulation. This multivesicular structure plays a vital role in its hypoglycemic effect. Although the outer liposomal membrane is destroyed due to high glucose stimulation, the internal structure of MVLs can remain intact to preserve insulin. As described in the literature, MVLs present enormous potential in drug delivery based on their unique structure [22,24].

In the in vitro release experiments (Figure 6), the insulin release rate from MVLs in the 100 mg/dL glucose solution was marginally reduced when compared with the glucose-free solution. The difference between the two release curves could be attributed to the protective effect of glucose enrichment. In the 100 mg/dL glucose solution, the generated gluconic acid and H_2_O_2_ are not sufficient to destroy liposomal membranes. The glucose enrichment on the surface of MVLs prevents insulin release. From 100 to 400 mg/dL, the insulin release rate increased with increasing glucose concentrations. In the 200 and 400 mg/dL glucose solutions, the local reduced pH and generated H_2_O_2_ destroyed liposomal membranes and triggered insulin release. This effect is strong when compared with the protective effect of glucose enrichment, thus accelerating insulin release. Notably, this insulin release behavior is of considerable significance for the effective regulation of BGLs.

The trigger mechanism based on in situ catalysis affords the basis for the controlled release of insulin in MVLs. The selection of PBA derivatives is a critical factor in achieving in situ catalysis. Following the screening process, FOP, possessing a fluorinated benzene ring and an eight-carbon aliphatic chain, was selected to participate in the construction of the glucose-responsive systems. In this work, the pKa value of FBP (6.79) was lower than that of BP (7.53), and the pKa value of FOP (6.90) was lower than that of OP (7.84). The pKa values of fluorinated compounds were lower than that of non-fluorinated compounds, consistent with previously reported findings in the literature [44]. The introduction of an electron-withdrawing fluorine atom can reduce the pKa value of OP. Therefore, FOP has a higher affinity to glucose than OP. In addition, the EE of FOP (18.75 ± 2.50%) was higher than that of FBP (3.10 ± 0.52%); this could be explained by the lipid solubility of FOP when compared with FBP. The longer the alkyl chain of PBA derivatives, the better the lipid solubility of PBA derivatives [45]. The alkyl chain of FOP has eight carbon atoms, whereas FBP has only four carbon atoms. Accordingly, the eight-carbon aliphatic chain confers high lipid solubility on FOP. FOP in the liposomal membrane binds with glucose through reversible boronate formation, resulting in glucose enrichment on the MVL surface (Figure 7). Previous studies have confirmed that the ortho-hydroxyl group of glucose can form a complex with PBA [46]. Glucose cannot be observed under an optical microscope; thus, the Alizarin red containing ortho-hydroxyl group was introduced to confirm the interaction between 1,2-diols and the MVL membrane. GOx outside MVLs is slowly metabolized by lymphatic capillaries owing to its high molecular weight (approximately 150 kDa) and can accumulate subcutaneously for a sufficient period to induce catalysis [47,48]. Moreover, we identified that GOx and MVLs interacted via hydrophobic interactions and hydrogen bonds, which were confirmed by fluorescence experiments and molecular docking, respectively. The interactions between GOx and MVLs are also beneficial for in situ catalysis. Notably, a molecular docking method for analyzing the interaction between GOx and liposomal membranes was proposed in the present study.

In situ, GOx catalyzes the rapid and efficient conversion of glucose into gluconic acid and H_2_O_2_, and the local acidic and H_2_O_2_-enriched microenvironment affords the basis for the glucose responsiveness of MVL(F+E+I). Therefore, the pH and H_2_O_2_ sensitivities of MVL(F+E+I) render it glucose-responsive. In the present study, the dual sensitivity of MVL(F+E+I) was confirmed by in vitro release tests. Almost all the insulin was released at pH 4.0 in 1 h, whereas only a small amount of insulin was released at pH 7.4 (Figure 8A). The pH sensitivity of MVL(F+E+I) is conferred by DSPE in the membrane, and the protonation of DSPE leads to lipid membrane instability [43,49]. Notably, the pH-sensitive MVLs were obtained for the first time. This pH-sensitive MVL could be employed as a carrier to deliver various therapeutic agents for disease treatment. Moreover, the insulin release rate of MVL(F+E+I) increased with increasing H_2_O_2_ concentrations (Figure 8B). The local H_2_O_2_-enriched environment can induce lipid peroxidation [50,51]. Then, the permeability and stability of liposomal membranes are modified, which leads to the destruction of liposomal structures.

In animal studies, MVL(F+E+I) exhibited excellent regulation of BGLs, good resistance to glucose challenge, and low hypoglycemic index (Figure 9). MVL(F+E+I) showed a better antidiabetic efficacy than MVL(F+I) or MVL(E+I) (Figure 9A). BGLs were decreased gradually in the MVL(E+I) group when compared with the MVL(F+E+I) group; this can be explained by the lack of in situ catalysis of GOx based on FOP in the MVL(E+I) group. The addition of FOP induced glucose enrichment on the surface of MVL(F+E+I). Given the glucose enrichment, MVL(F+E+I) quickly responded to glucose to release insulin under hyperglycemic conditions. In the MVL(F+I) group, BGLs steadily increased after 4 h, as MVL(F+I) could not provide sufficient insulin after the initial burst release. In contrast, BGLs in the MVL(F+E+I) group were maintained within the normoglycemic range (< 200 mg/dL) after an initial reduction in BGLs. The acidic and H_2_O_2_-enriched microenvironment surrounding the MVL(F+E+I) membrane could be formed under hyperglycemic conditions due to the addition of enzymes, and BGLs could be reduced and maintained within the normal range. Therefore, FOP and biological enzymes play a crucial role in the glucose responsiveness of MVLs; this is in accordance with the original hypothesis. The peak serum insulin level could be achieved in less than 1 h following a subcutaneous insulin injection (Figure 9E); our finding is consistent with previous studies [32]. For MVL(F+E+I), the peak serum insulin level was reached in approximately 2 h (Figure S17). Under hyperglycemic conditions, sufficient insulin released from MVL(F+E+I) reduced BGLs to the normoglycemic level. Accordingly, the peak serum insulin level was delayed following MVL(F+E+I) treatment when compared with the insulin solution. Moreover, when blood glucose decreased to the normal level, insulin release was reduced to maintain BGLs in the normoglycemic state. Finally, we determined the cytotoxicity of PBA derivatives and inflammatory reaction of MVL(F+E+I), and the results confirmed the safety of MVL(F+E+I) (Figure 10). In comparison with other reported systems, composed of synthetic materials, MVLs are both biodegradable and biocompatible, since their components are mainly derived from naturally occurring lipids.

Our research still has several limitations. Further research is crucial for optimizing the sensitivity of the glucose response and establishing long-term animal experiments assessing MVLs. Moreover, the docking model of MVLs needs to be constructed and used to analyze the interaction of MVLs and proteins. Despite these limitations, this MVL with its modified membrane offers a clinical opportunity for glucose-responsive insulin delivery and is expected to maintain blood glucose homeostasis precisely to reduce the complications in patients with diabetes.

## 5. Conclusions

In summary, a new glucose-responsive insulin delivery strategy was explored by formulating MVLs sensitive to both pH and H_2_O_2_. The glucose-responsive MVLs, prepared by the double emulsion method, contain FOP in the membrane and can adsorb glucose onto the MVL surface. The local acidic and H_2_O_2_-enriched microenvironment is further induced by the in situ catalysis of GOx under high glucose conditions; this destroys the outer MVL membrane and triggers insulin release. In vitro experiments revealed that the glucose-responsive MVLs could judiciously control insulin release in response to fluctuating glucose concentrations. Moreover, the stable membrane structure of MVL(F+E+I) was achieved by the addition of FOP in the present study. According to in vivo experiments, the formulated glucose-responsive MVLs could be highly effective in reducing BGLs and maintaining normoglycemia. Overall, biocompatible MVLs offer a promising strategy for glucose-responsive insulin delivery and provide an effective approach for the management of diabetes.

## Figures and Tables

**Table 1:** Physicochemical properties of multivesicular liposomes with different molar ratios of DOPC to DSPE. Data are shown as mean ± SD (*n* = 3).

Formulation ID	Molar Ratio of DOPC to DSPE	Appearance	EE of Insulin (%)	Structure of MVLs in PBS (pH 4.0) for 1 h
F1	0:1	Aggregated	−	−
F2	1:1	Uniform	37.24 ± 2.52	−
F3	3:1	Uniform	49.79 ± 1.50	Broken
F4	4:1	Uniform	63.08 ± 2.92	Intact

− Indicates that the experiment was not performed.

## Data Availability

The data presented in this study are available on request from the corresponding author.

## Supplementary Materials

The following are available online at https://www.mdpi.com/article/10.3390/pharmaceutics14010021/s1, Figure S1: Schematic of enzymatic reactions that involve GOx and CAT, Figure S2: ESI-MS spectrum of EP, Figure S3: ESI-MS spectrum of BP, Figure S4: ESI-MS spectrum of FBP, Figure S5: ESI-MS spectrum of OP, Figure S6: ESI-MS spectrum of FOP, Figure S7: ^1^H NMR spectrum of BP (300 MHz in CHCl_3_), Figure S8: ^1^H NMR spectrum of FBP (300 MHz in CHCl_3_), Figure S9: ^1^H NMR spectrum of OP (300 MHz in CHCl_3_), Figure S10: ^1^H NMR spectrum of FOP (300 MHz in CHCl_3_), Figure S11: Particle size changes in MVL(F+E+I) in PBS (pH 7.4) with different glucose concentrations, Figure S12: In vitro accumulated insulin release from MVL(E+I) in PBS (pH 7.4) with different glucose concentrations, Figure S13: Docking study of the interaction of DOPC with GOx, Figure S14: Docking study of the interaction of DPPG with GOx, Figure S15: Docking study of the interaction of FOP with GOx, Figure S16: Docking study of the interaction of cholesterol with GOx, Figure S17: Blood glucose and serum insulin levels of diabetic rats treated with MVL(F+E+I) in 10 h, Table S1: Insulin leakage rate from MVL(F+E+I) in a month.
