# Supplementary material for: Multivesicular Liposomes for Glucose-Responsive Insulin Delivery
Source: Pharmaceutics. 2021 Dec 22;14(1):21. doi: 10.3390/pharmaceutics14010021 (PMC8781467; doi:10.3390/pharmaceutics14010021)
Supplement: Supplementary file 1 [file pharmaceutics-14-00021-s001.zip › pharmaceutics-1491968-supplementary.pdf]

pharmaceutics

Supplementary Materials: Multivesicular liposomes for glucose-responsive insulin delivery

Guangqu Liu, Suping He, Yu Ding, Cai Chen, Qingchun Cai and Wei Zhou

1. Characterization of PBA derivatives

1.1. EP

White solid; yield 87.0%. HRMS (ESI-TOF) *m*/*z*: calcd. for $C_9H_{11}BO_4$ $[M-H]^-$ 193.0750; found 193.0684.

1.2. BP

White solid; yield 76.6%. HRMS (ESI-TOF) *m*/*z*: calcd. for $C_{11}H_{15}BO_4$ $[M-H]^-$ 221.1063; found 221.0991. ^{1}H NMR (300 MHz, $CDCl_3$): δ 8.32–8.33 (d, *J* = 3 Hz, 1H, Ar-H), 8.19–8.20 (d, *J* = 3 Hz, 1H, Ar-H), 8.07–8.08 (d, *J* = 3 Hz, 1H, Ar-H), 7.84–7.85 (d, *J* = 3 Hz, 1H, Ar-H), 5.32 (s, 2H, OH), 4.35–4.42 (m, 2H, OCH_2), 1.76–1.85 (m, 2H, CH_2), 1.47–1.58 (m, 2H, CH_2), 0.99–1.05 (m, 3H, CH_3).

1.3. FBP

White solid; yield 84.9%. HRMS (ESI-TOF) *m*/*z*: calcd. for $C_{11}H_{14}BFO_4$ $[M-H]^-$ 239.0969; found 239.0896. ^{1}H NMR (300 MHz, $CDCl_3$): δ 8.03–8.09 (m, 1H, Ar-H), 7.93–7.96 (m, 1H, Ar-H), 7.53–7.59 (m, 1H, Ar-H), 5.32 (s, 2H, OH), 4.35–4.43 (m, 2H, OCH_2), 1.75–1.84 (m, 2H, CH_2), 1.48–1.55 (m, 2H, CH_2), 0.99–1.04 (m, 3H, CH_3).

1.4. OP

White solid; yield 67.8%. HRMS (ESI-TOF) *m*/*z*: calcd. for $C_{15}H_{23}BO_4$ $[M-H]^-$ 276.1689; found 277.1622. ^{1}H NMR (300 MHz, $CDCl_3$): δ 8.35–8.36 (d, *J* = 3.0 Hz, 2H, Ar-H), 8.22–8.23 (d, *J* = 3.0 Hz, 2H, Ar-H), 4.41–4.43 (m, 2H, OCH_2), 1.80–1.89 (m, 2H, CH_2), 1.50–1.55 (dd, 2H, CH_2), 1.33–1.44 (m, 8H, $CH_2CH_2CH_2CH_2$), 0.93–0.96 (t, *J* = 3.0 Hz, 3H, CH_3).

1.5. FOP

White solid; yield 77.7%. HRMS (ESI-TOF) *m*/*z*: calcd. for $C_{15}H_{22}BFO_4$ $[M-H]^-$ 295.1595; found 295.1519. ^{1}H NMR (300 MHz, $CDCl_3$): δ 8.06–8.12 (m, 1H, Ar-H), 7.96–7.99 (m, 1H, Ar-H), 7.55–7.61 (m, 1H, Ar-H), 4.37–4.44 (m, 2H, OCH_2), 1.79–1.88 (m, 2H, CH_2), 1.45–1.55 (m, 2H, CH_2), 1.35–1.44 (m, 8H, $CH_2CH_2CH_2CH_2$), 0.92–0.96 (m, 3H, CH_3).

2. Supplementary figures

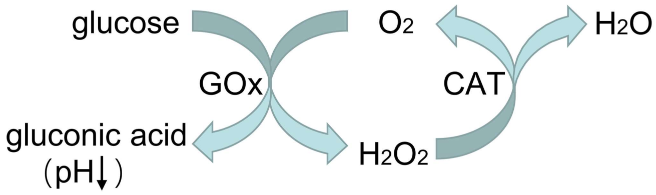

Figure S1. Schematic of enzymatic reactions that involve GOx and CAT. CAT, catalase.

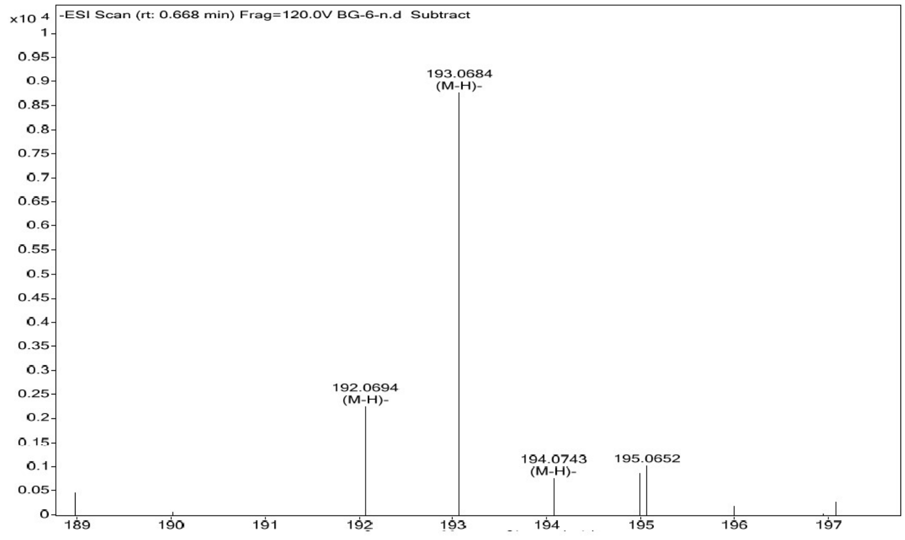

Figure S2. ESI-MS spectrum of EP.

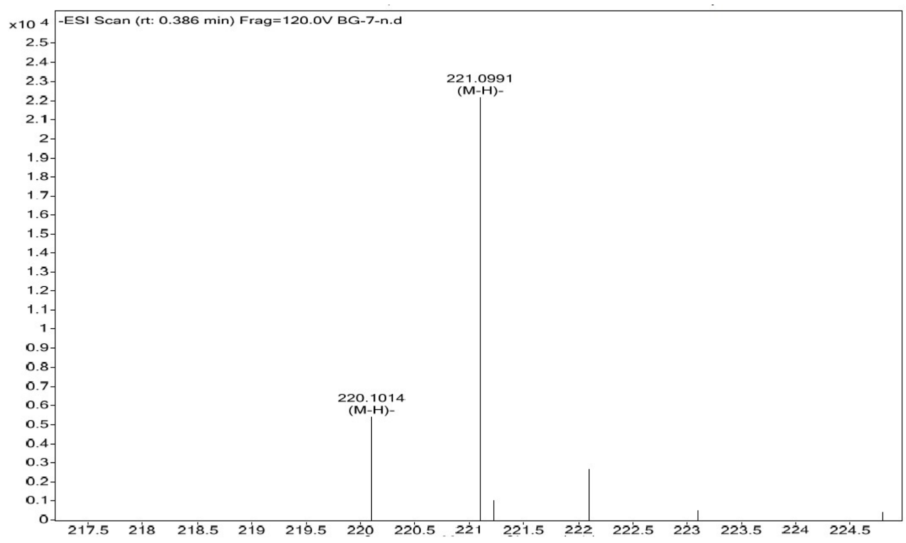

Figure S3. ESI-MS spectrum of BP.

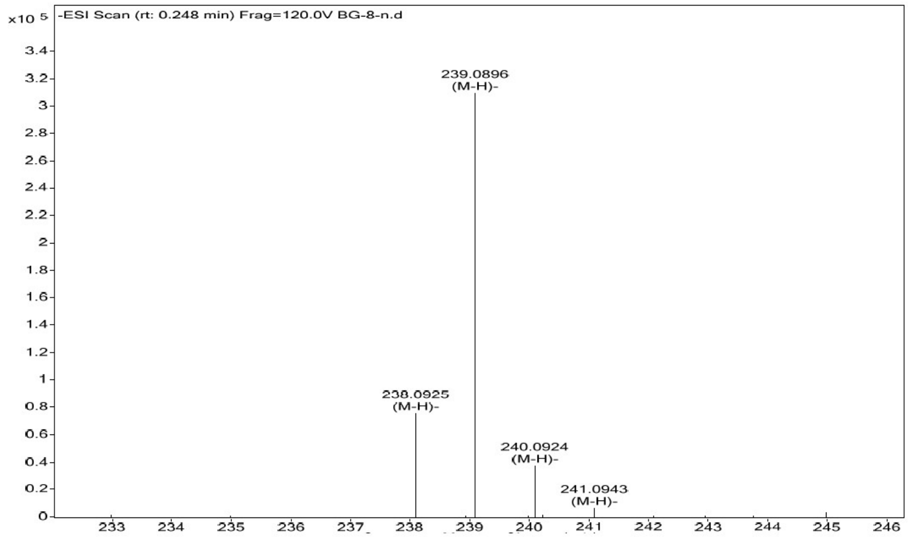

Figure S4. ESI-MS spectrum of FBP.

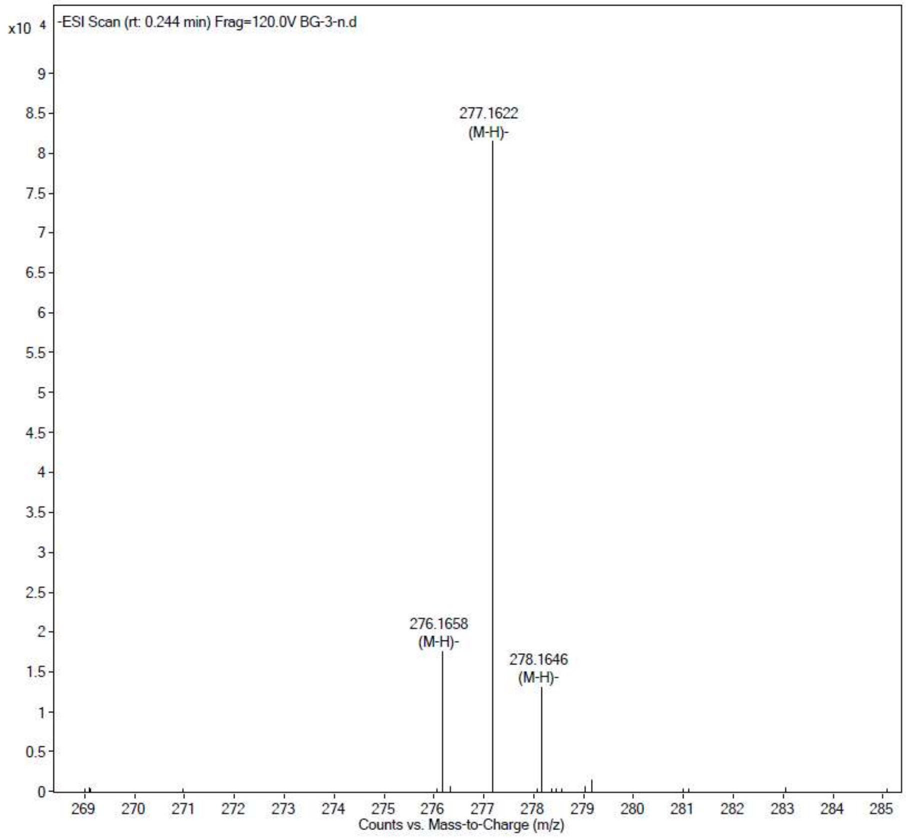

Figure S5. ESI-MS spectrum of OP.

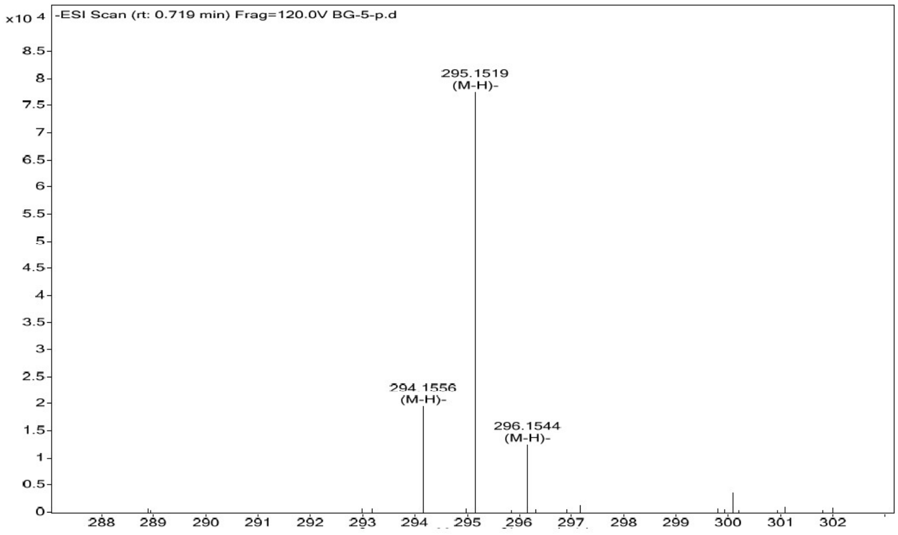

Figure S6. ESI-MS spectrum of FOP.

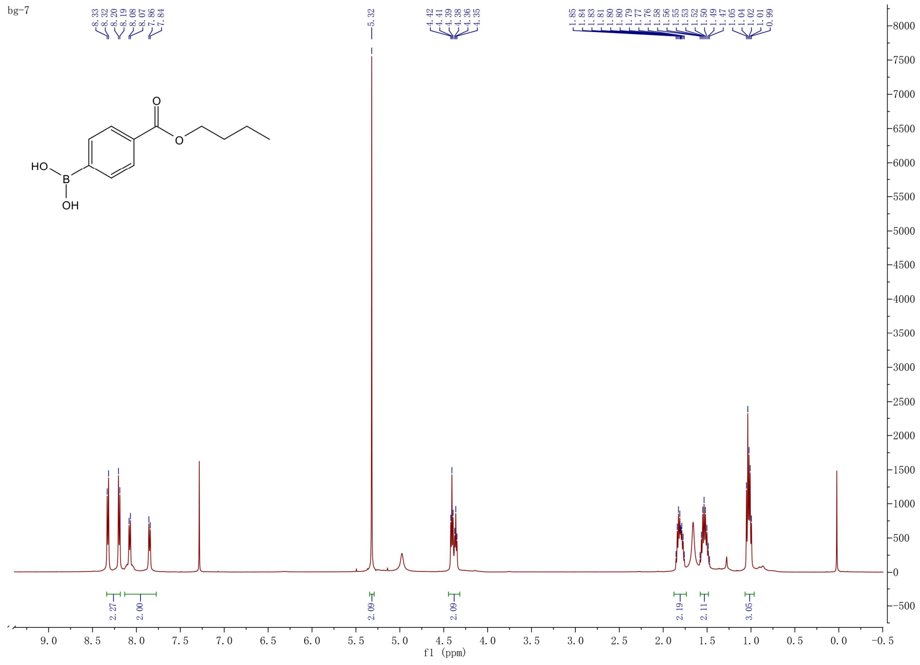

Figure S7. ^{1}H NMR spectrum of BP (300 MHz in $CHCl_3$).

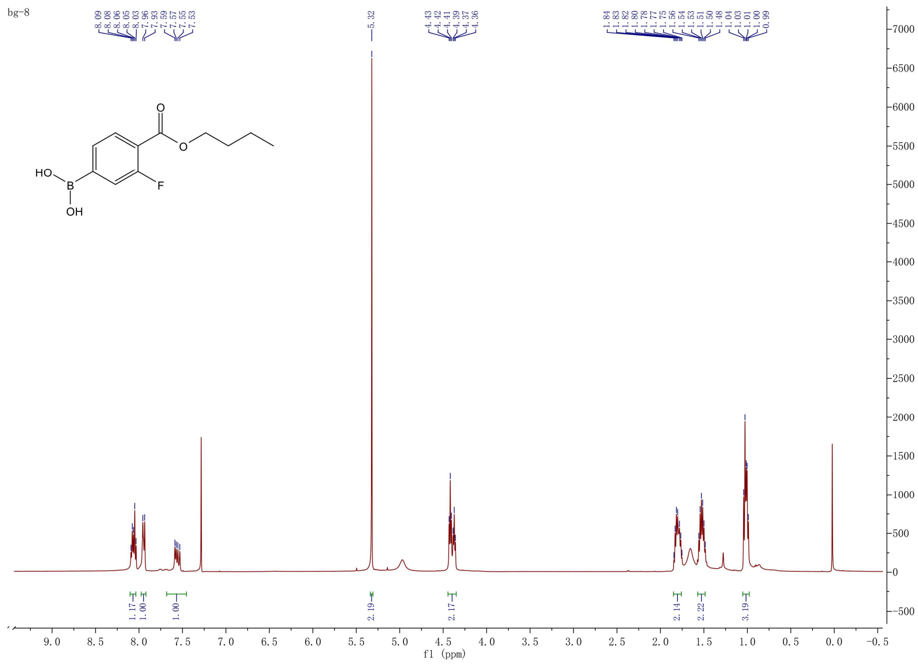

Figure S8. ^{1}H NMR spectrum of FBP (300 MHz in $CHCl_3$).

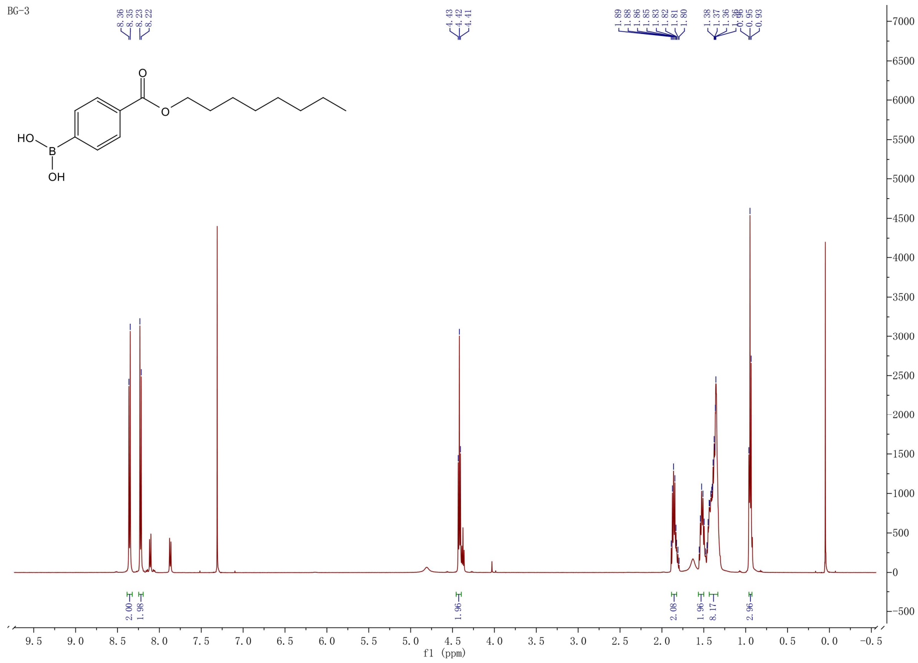

Figure S9. ^{1}H NMR spectrum of OP (300 MHz in $CHCl_3$).

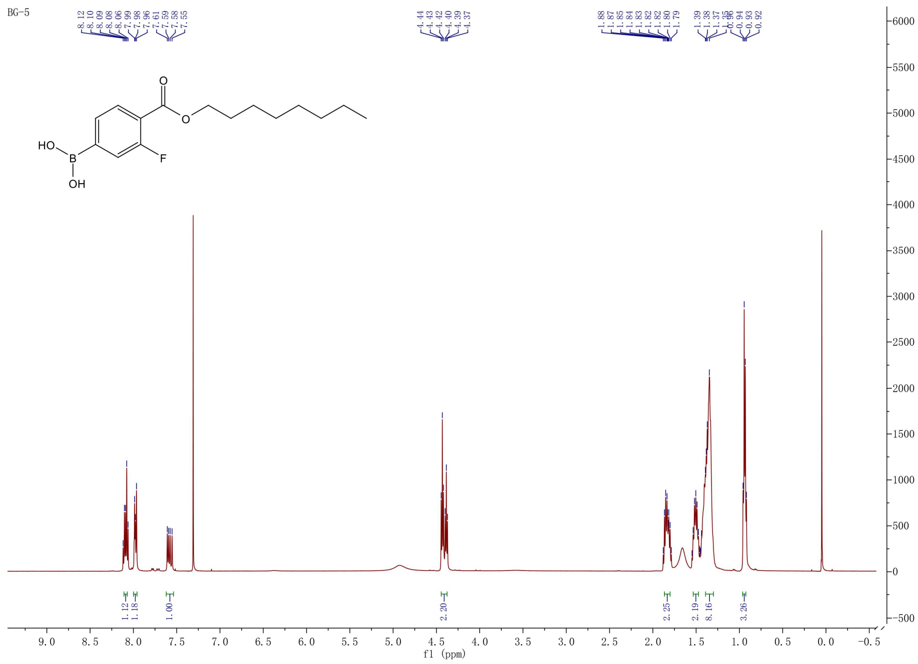

Figure S10. ^{1}H NMR spectrum of FOP (300 MHz in $CHCl_3$).

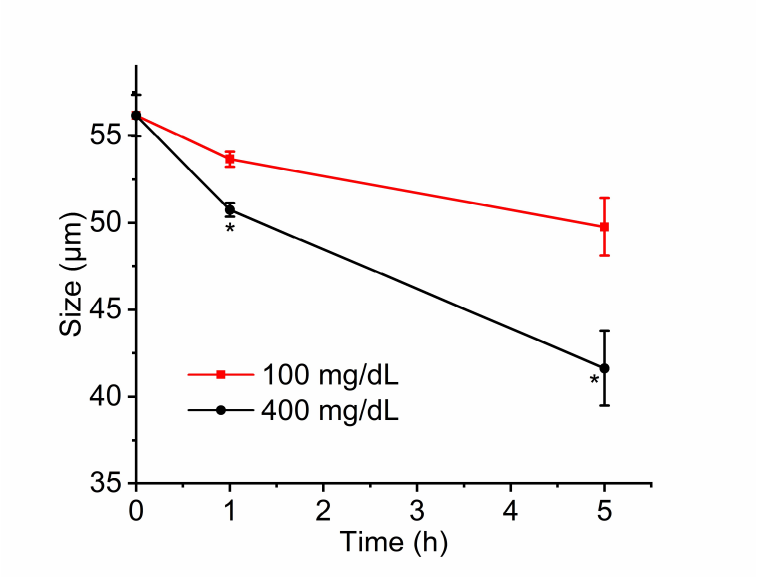

Figure S11. Particle size changes in MVL(F+E+I) in PBS (pH 7.4) with different glucose concentrations. Statistical significance was evaluated by a two-tailed, unpaired t-test (* $p < 0.05$). Data are shown as mean ± SD ($n = 3$).

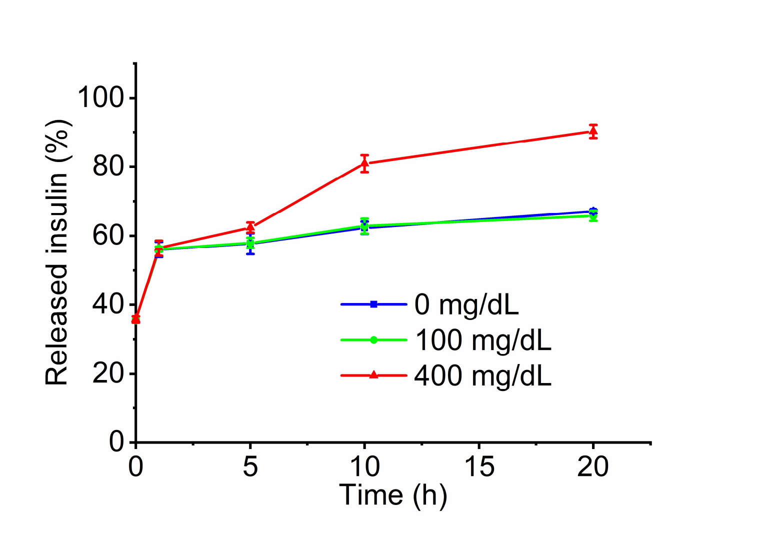

Figure S12. In vitro accumulated insulin release from MVL(E+I) in PBS (pH 7.4) with different glucose concentrations. Data are shown as mean ± SD (n = 3).

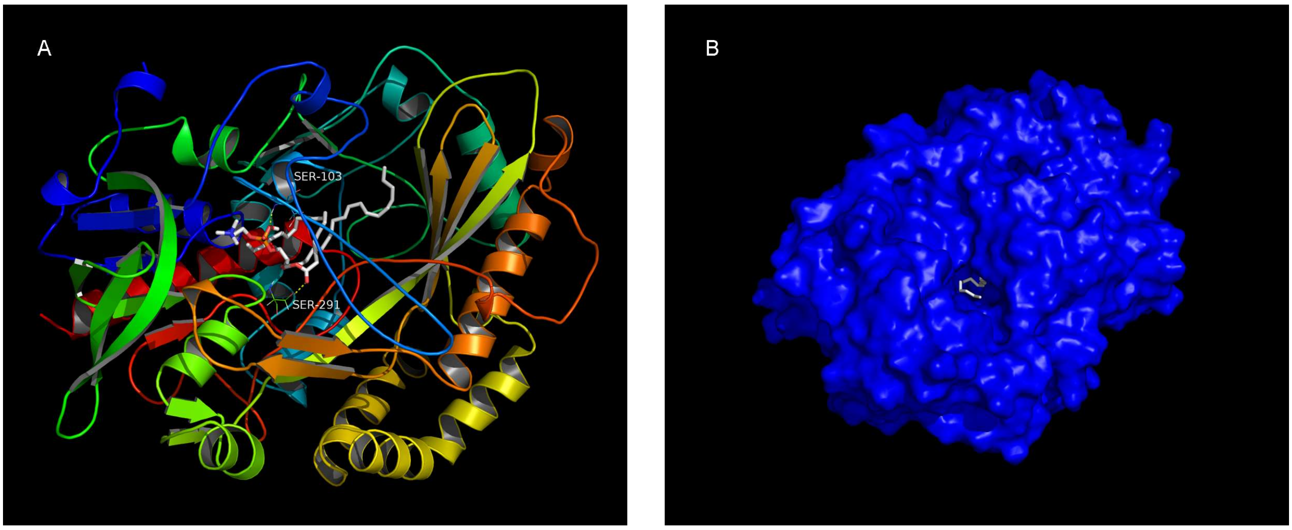

Figure S13. Docking study of the interaction of DOPC with GOx. DOPC is shown as sticks. (A) Residues involved in the interaction with DOPC are labeled and shown as lines, with the remainder of GOx shown as a cartoon. Hydrogen bonds are indicated by yellow doted lines. (B) GOx is shown as a surface.

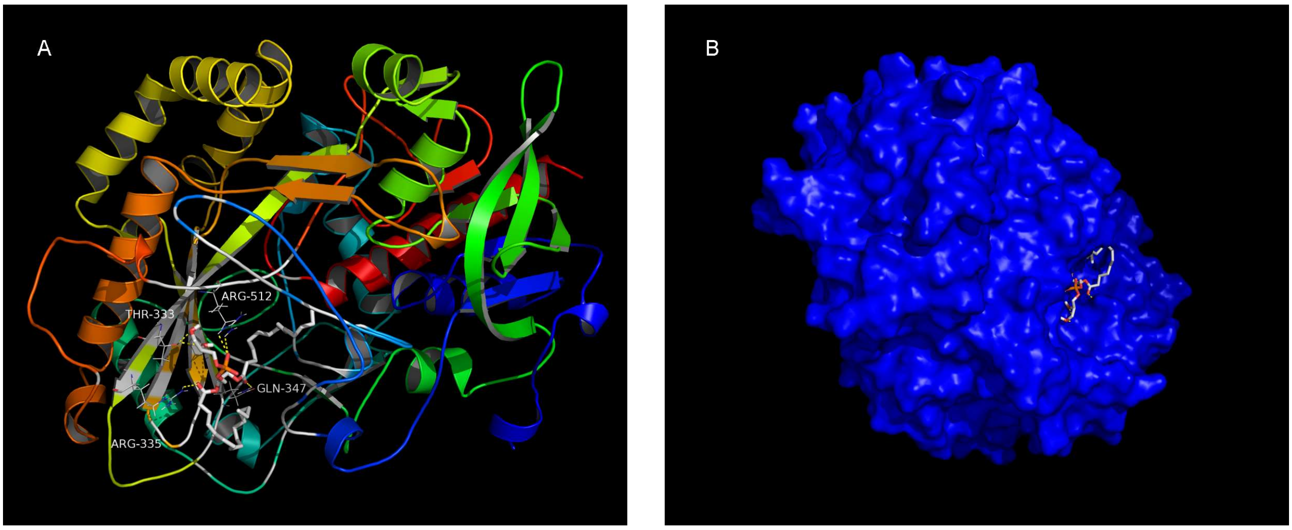

Figure S14. Docking study of the interaction of DPPG with GOx. DPPG is shown as sticks. (A) Residues involved in the interaction with DPPG are labeled and shown as lines, with the remainder of GOx shown as a cartoon. Hydrogen bonds are indicated by yellow doted lines. (B) GOx is shown as a surface.

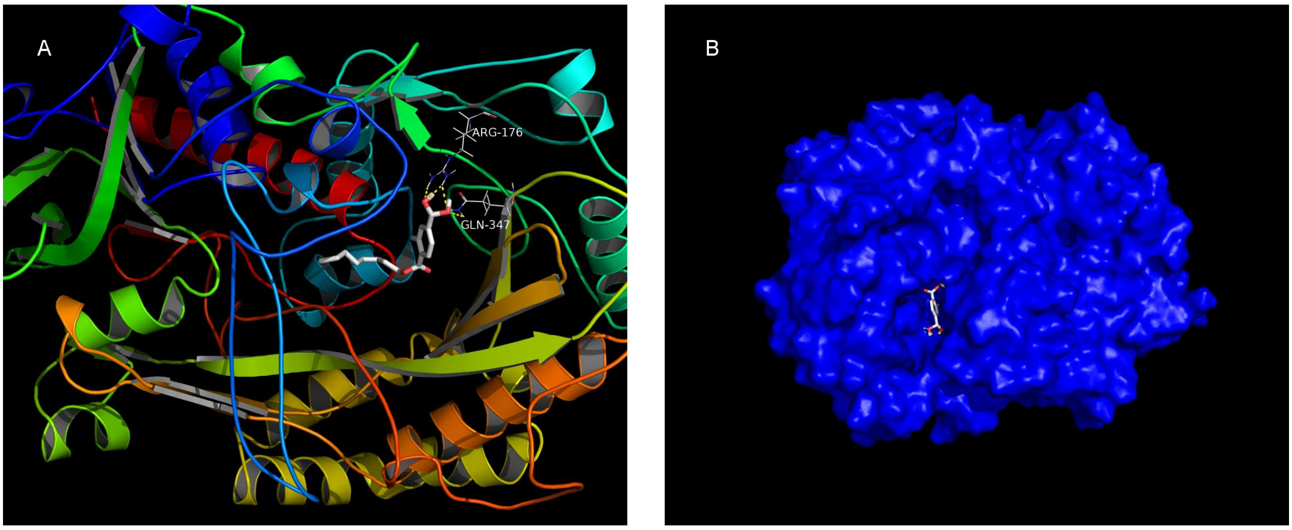

Figure S15. Docking study of the interaction of FOP with GOx. FOP is shown as sticks. (A) Residues involved in the interaction with FOP are labeled and shown as lines, with the remainder of GOx shown as a cartoon. Hydrogen bonds are indicated by yellow doted lines. (B) GOx is shown as a surface.

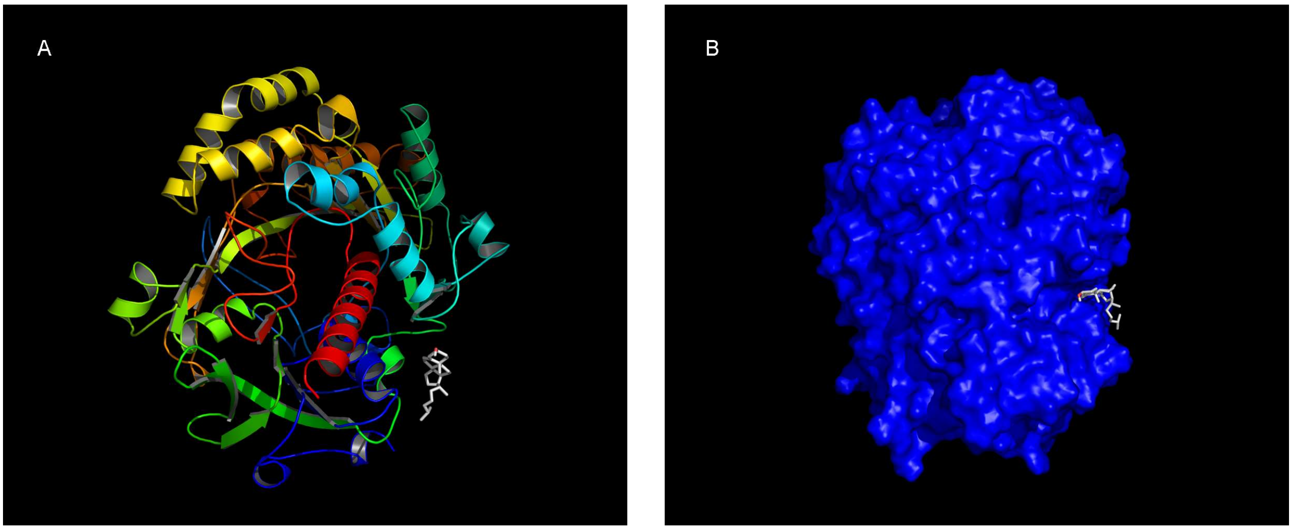

Figure S16. Docking study of the interaction of cholesterol with GOx. Cholesterol is shown as sticks. GOx is shown as a cartoon (A) or a surface (B).

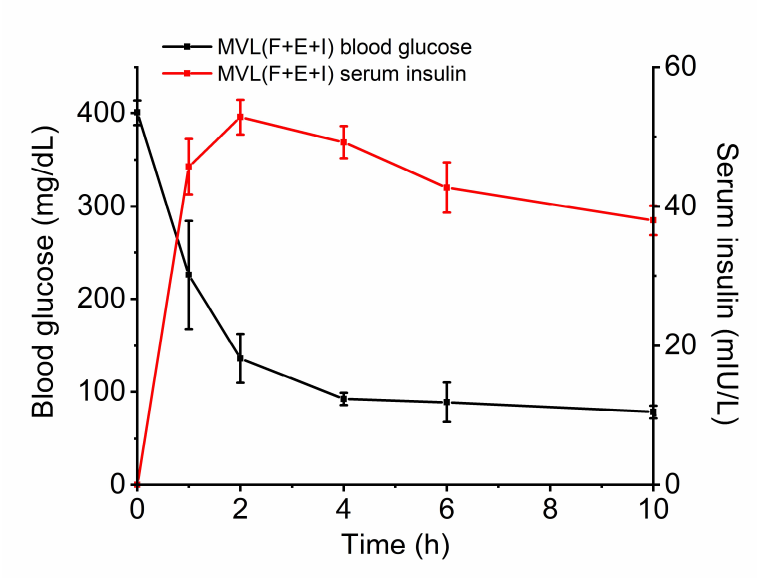

Figure S17. Blood glucose and serum insulin levels of diabetic rats treated with MVL(F+E+I) in 10 h. Data are shown as mean ± SD (n = 5).

3. Supplementary table

Table S1. Insulin leakage rate from MVL(F+E+I) in a month. Data are shown as mean ± SD (n = 3).

**Time**	3 days	1 week	4 weeks
**Insulin leakage rate (%)**	2.19 ± 0.27	3.27 ± 0.49	3.26 ± 0.16
